# Selective Inhibition of Tumor Necrosis Factor for Attenuating Alzheimer’s Disease: Strategies Targeting Neuroinflammation

**DOI:** 10.1007/s10753-026-02492-9

**Published:** 2026-03-14

**Authors:** Janakiraman Pillai Udaiyappan, Rengasamy Balakrishnan, Manivasagam Muthukumaran Tamilarasan, Balamuralikrishnan Balasubramanian, Kasim Sakran Abass

**Affiliations:** 1 Department of Biological Sciences, Southern Methodist University, Dallas, TX 75275 USA; 2 Department of Biotechnology, College of Biomedical and Health Science, Research Institute of Inflammatory Disease (RID), Konkuk University, Chungju, 27478 South Korea; 3 Department of Biochemistry and Biotechnology, Annamalai University, Annamalai Nagar, Tamil Nadu 608 002 India; 4 Department of Food Science and Biotechnology, College of Life Science, Sejong University, Seoul, 05006 Republic of Korea; 5 Department of Physiology, Biochemistry, and Pharmacology, College of Veterinary Medicine, University of Kirkuk, Kirkuk, 36001 Iraq

**Keywords:** Alzheimer’s disease, Neuroinflammation, TNF inhibitors, Etanercept, Infliximab, Adalimumab, And XPro1595

## Abstract

Neuroinflammation is increasingly recognized as a key feature in the development of Alzheimer’s disease (AD), with tumor necrosis factor-alpha (TNF-α) playing a crucial role in initiating inflammatory responses. Continuous activation of TNF-α leads to synaptic dysfunction, neuronal loss, and worsening of amyloid and tau pathology. Specifically, the upregulation of the pro-inflammatory cytokine TNF-α in the brain activates its receptors (TNFR1 & TNFR2). Targeting TNF-α through selective inhibition presents a promising therapeutic strategy for regulating neuroinflammatory responses without compromising systemic immunity. This review discusses current insights into TNF-α signaling in AD progression and examines the effectiveness of selective TNF-α inhibitors in preclinical and clinical studies. We also highlighted specific TNF-α inhibitors, including small molecules and gene therapy approaches, for chronic inflammatory conditions and discussed the limitations and future directions of the current review. Targeted TNF-α inhibition could serve as a novel, disease-modifying treatment for AD, especially when combined with multi-targeted approaches addressing amyloid burden, tau pathology, oxidative stress, and neuroinflammation.

## Introduction

The World Health Organization (WHO) projected that there are approximately 46.8 million cases of Alzheimer's disease (AD) worldwide, and this number is expected to double every 20 years, with an estimated total of 131.5 million cases in 2050 [1]. AD affects nearly 10% of people aged 65 and older and over 30% of those aged 85 and older worldwide, and it is known to be the leading cause of dementia (∼70% of cases) [2]. AD is a devastating neurodegenerative disorder characterized by progressive cognitive decline and is frequently accompanied by comorbid symptoms including amnesia, mood and behavioral disturbances, disorientation, language impairment, and, in later stages, motor dysfunction [3–5]. In the pathogenesis of AD, Aβ plays a key driver in triggering numerous pathophysiological processes, including oxidative stress, synaptic plasticity impairment, and apoptosis, which are key contributors to cognitive decline [6]. While genetic, environmental, and intrinsic factors are recognized as contributors to the onset and progression of AD [7]. The precise pathological mechanisms remain unclear. This limited understanding has hindered progress in developing and approving therapies that can alter the course of the disease.

Interestingly, clinical trials (Clinical trial number: not applicable) targeting the removal of senile plaques in AD patients have yielded mixed outcomes. While reductions in protein aggregate volume were observed in some cases, this clearance did not consistently translate into cognitive improvement [6, 7]. More recent investigations, however, indicate that monoclonal antibody therapies such as aducanumab, lecanemab, and the newer donanemab can markedly slow disease progression in the early stages of AD and can eliminate up to 90% of amyloid deposits. Eli Lilly, the developer of donanemab, reported promising findings from a large trial involving 1,736 participants, presented on July 17, 2023, at the Alzheimer’s Association International Conference in Amsterdam [8]. Their data revealed that 47% of patients receiving donanemab showed no measurable cognitive decline, compared with only 29% in the placebo group.

Despite these encouraging results, amyloid-related imaging abnormalities (ARIA) remain a significant adverse effect of antibody-based therapies. The ARIA is found as vasogenic edema or effusion (ARIA-E) and hemosiderin-related changes (ARIA-H) on Magnetic Resonance Imaging (MRI) [9]. In Eli Lilly’s Phase III trial, approximately one-quarter of patients developed ARIA, with three fatalities linked to the condition [8]. Since ARIA monitoring is both costly and complex, there is a pressing need to better understand the role of neuroinflammation in AD progression. Furthermore, research leaders and developers emphasize that combination therapy may offer the greatest potential for treating such a multifaceted disorder. Drawing parallels to cardiovascular medicine, where combinations of antithrombotics, antihypertensives, and lipid-lowering drugs have substantially improved patient outcomes, they highlight the possibility that neuroinflammation inhibitors could both mitigate Alzheimer’s risks and enhance the benefits of antibody-based treatments.

## Neuroinflammation and AD

Normal brain function, including processes such as cognition and behavior, relies on healthy communication between neurons, glial cells, soluble mediators, and the immune system. Although the Central Nervous System (CNS) has long been considered an immune-privileged site due to its protective barriers, it is not fully isolated from the peripheral immune system and can respond to signals of injury or dysfunction from peripheral organs [10]. Neuroinflammation refers to an inflammatory response within the CNS that may arise from internal pathological events (such as ischemia, cellular stress, or molecular signals) or external factors (including infections, trauma, or toxins) [11]. This process is characterized by the activation of microglia and astrocytes, increased permeability of endothelial cells, and the infiltration of blood-derived immune cells following biochemical or structural damage to brain tissue or the blood–brain barrier (BBB) [12]. During the neuroinflammatory process, microglia get over-activated and oversensitive, and they can trigger the release of several cytotoxic molecules like tumor necrosis factor (TNF), pro-inflammatory cytokines (e.g., IL-1β, IL-6, IL-18), and interferon-γ (IFN-γ), and oxidative metabolites (e.g., reactive nitrogen and oxygen species, and nitric oxide) [13]. TNF is the sole and key promoter of inflammation in several chronic inflammatory and degenerative diseases [14–16], including AD [16, 17].

**Fig. 1:**
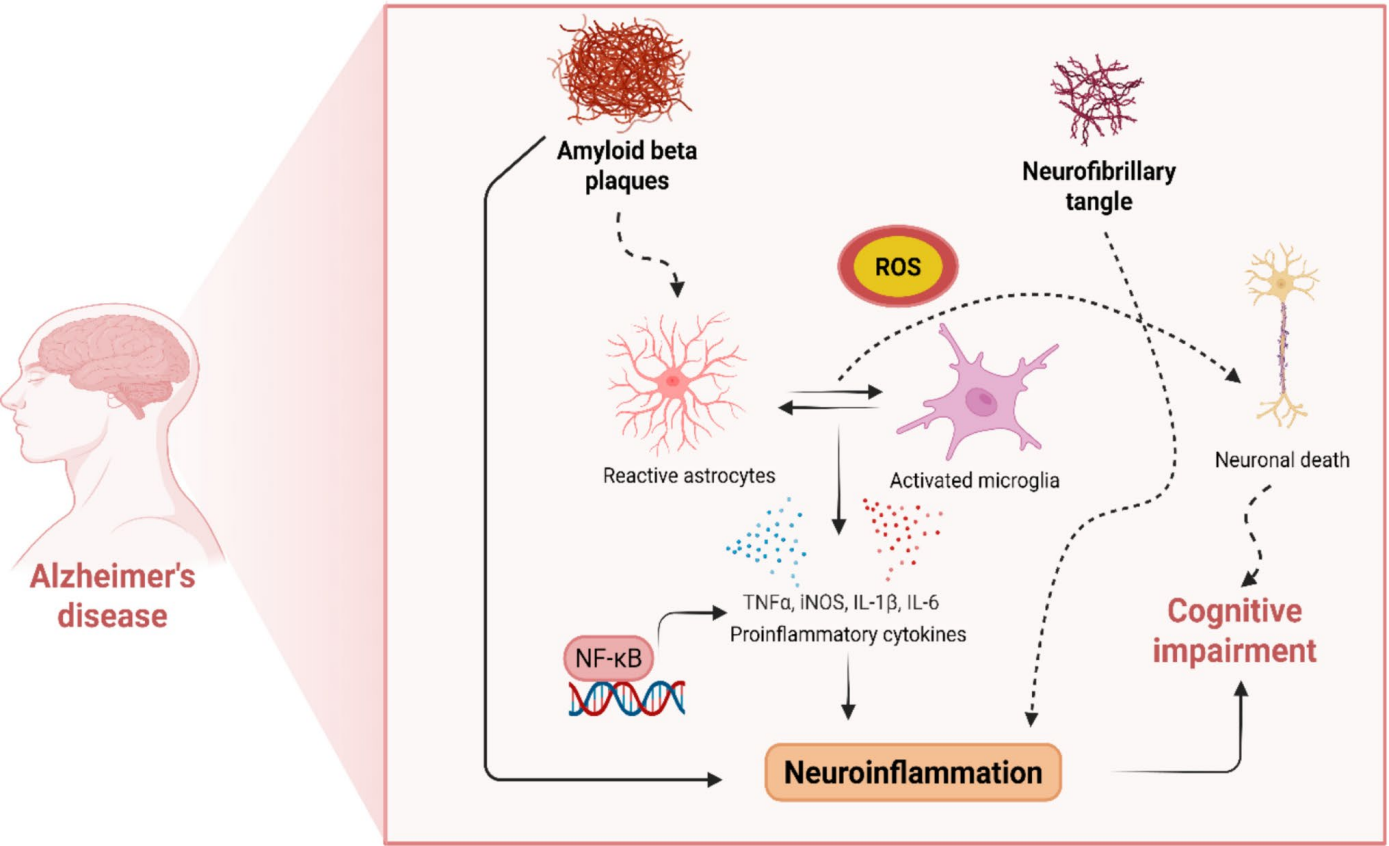
Neuroinflammation in the pathogenesis of Alzheimer's disease. AD is characterized by sustained activation of microglia and astrocytes in response to amyloid-β (Aβ) plaques and tau tangles. Although these glial cells initially exert protective effects by clearing Aβ, prolonged stimulation drives them toward a chronic proinflammatory phenotype. This maladaptive activation results in excessive release of inflammatory cytokines and mediators, causing neuronal injury and cognitive impairment. Created with BioRender.com

Furthermore, neuroinflammation has been reported to contribute to AD pathogenesis [18, 19]. Due to its ability to enhance the deposition of Aβ and tau proteins [20]. Abnormal Aβ deposition then triggers downstream activation of the inflammatory response. Astrocytes and microglia were recruited and activated by Aβ, leading to the secretion of several chemokines [21, 22]. The worsening of neuroinflammation could induce neuronal degeneration and further enhance Aβ accumulation. Pacoal et al. indicated that microglial activation is the key factor for the deposition of tau tangles in the neocortex. They demonstrated that Aβ pathology could support tau deposition in the neocortex by inducing microglial activation, suggesting a triangular relationship among Aβ, tau, and microglial activation in AD [23]. In addition, AD patients' cognitive decline and disease progression are linked to acute and chronic inflammation [24–26] (Fig. 1).

Neurotoxic processes, synaptic impairment, and reduced neurogenesis are largely driven by proinflammatory mediators released in the injured CNS [27]. Elevated levels of TNF and IL-1β, in part through the release of prostaglandin E2, contribute to excitotoxicity and synaptic loss [28, 29]. In addition, numerous molecules, including macrophage-recruiting proteins, complement system components (such as C1q, C3b, C3c, C3d, and C4), coagulation factors, proteases, and pentraxins, play key roles in promoting neuronal dysfunction and degeneration [30]. Although much is known about the cellular and molecular mechanisms that shape AD pathology, a complete understanding is still lacking.

Astrocytes, which provide essential structural and metabolic support in the CNS, regulate neurotransmitter homeostasis, preserve BBB integrity, and supply nutrients to both mature and developing synapses [31]. They also clear cellular debris, amyloid, and tau aggregates, and respond to brain insults such as ischemia, infection, and abnormal protein deposition by forming scars and reactive gliosis [31]. The intermediate filament protein GFAP serves as a hallmark of astrocyte activation. Activated astrocytes can shift toward different phenotypes: the A1 type, induced via NF-κB signaling, produces proinflammatory mediators and can trigger neuronal apoptosis, whereas the A2 phenotype, driven by STAT3 activation, promotes anti-inflammatory and neuroprotective responses [32]. Whether astrocytes truly fall into these two distinct categories or exist along a functional continuum remains a matter of debate [31]. Evidence regarding their role in AD is also conflicting. On one side, astrocytes demonstrate the capacity to remove protein aggregates locally; however, studies of postmortem AD brains have revealed widespread populations of proinflammatory A1 astrocytes, suggesting impaired or maladaptive astrocytic responses [32–34]. Moreover, A1 astrocytes have been implicated in disrupting BBB function and cerebral blood flow, further contributing to AD onset and progression [31].

**Fig. 2:**
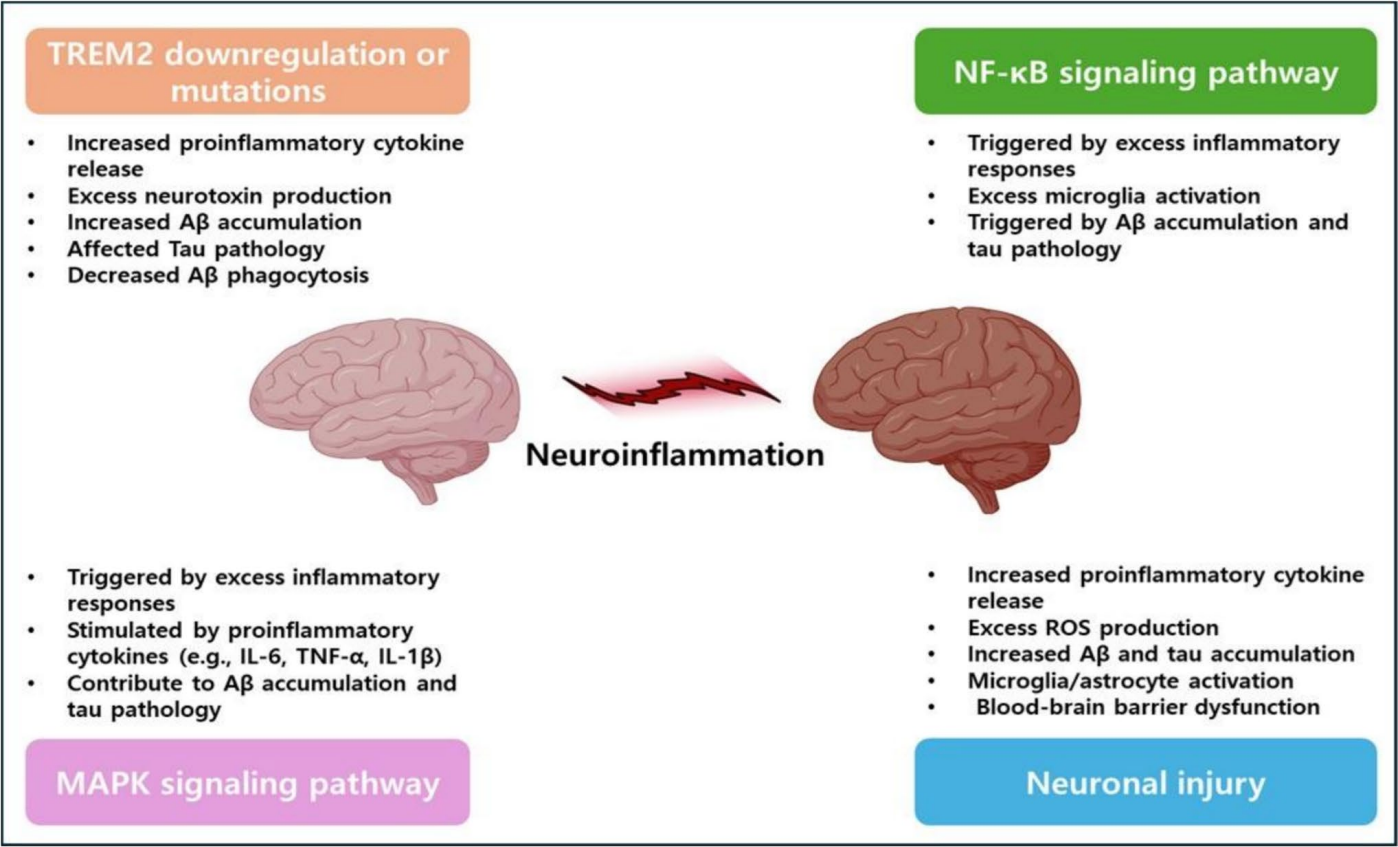
The primary molecular pathways driving neuroinflammation in AD are highly interconnected, collectively maintaining a state of chronic brain inflammation. Central to this network is neuroinflammation itself, which is primarily sustained by a few key pathways. First, Triggering Receptor Expressed on Myeloid Cells 2 (TREM2) downregulation leads to increased Aβ accumulation and tau phosphorylation, triggering the activation of neurotoxic astrocytes and microglia, which in turn cause neuronal damage. Second, the NF-κB pathway, activated by Aβ and tau, promotes the release of pro-inflammatory cytokines like TNF-α and IL-6, further fueling the inflammatory response. Third, neuronal injury induced by Aβ, reactive oxygen species (ROS), and mitochondrial dysfunction triggers the secretion of IL-1β and IL-18, amplifying neuroinflammation. Fourth, the activation of the MAPK pathway by Aβ, tau, and oxidative stress leads to increased ROS production and cytokine release, worsening oxidative and inflammatory damage. Together, these interconnected pathways form a complex network that underpins the persistent neuroinflammation characteristic of AD. Created with BioRender.com

Microglia, the brain's resident immune cells, are equally critical. They mediate phagocytosis of debris and pathogens, release signaling molecules to maintain tissue homeostasis, and regulate synaptic plasticity [35]. However, excessive cytokine levels in cerebrospinal fluid (CSF) have been shown to impair microglial clearance of Aβ [36]. Genetic studies have further revealed that rare mutations in the extracellular domain of TREM2 significantly increase AD risk, to a degree comparable to carrying the high-risk apolipoprotein E (APOE) ε4 allele [37]. TREM2 is highly expressed in microglia and plays a critical role in promoting phagocytosis [38–40]. However, excessive or prolonged stimulation can drive microglia into an abnormal state of activation, leading to changes in gene expression and morphology. In this state, microglia often assume an amoeboid form characterized by fewer processes and a reduced surveillance range [31]. The extent and persistence of external insults are closely linked to the degree of these morphological changes and the severity of tissue injury [31]. For instance, exposure to soluble hyperphosphorylated tau has been shown to alter microglial phenotype, impairing their normal physiological roles and promoting further accumulation of pathological protein aggregates [31, 41]. Traditionally, microglial activation has been described using the M1/M2 framework, in which M1 corresponds to a proinflammatory profile and M2 to an anti-inflammatory, tissue-repairing state [31]. Yet, similar to astrocytes, this binary classification may oversimplify reality; microglia are more likely to exist along a continuum of functional states, with overlapping features rather than two sharply defined phenotypes. During activation, microglia typically upregulate proteins such as TREM2, APOE, and TYRO protein tyrosine kinase-binding protein (TYROBP). In the early phases of neuroinflammation, gene expression tends to favor proliferation, whereas in later stages, immune-response genes become predominant, accompanied by the downregulation of homeostatic genes responsible for structural integrity, cell adhesion, and receptor function [31, 42, 43]. These dynamic transitions align with the heterogeneity observed in microglia isolated from postmortem brain tissue of AD patients [42]. Multiple pathways have been implicated in shaping these diverse activation states. Key contributors include NF-κB, mTOR, and MAPK signaling cascades; pro- and anti-inflammatory cytokines; complement proteins; chemokines; caspases; prostanoids; reactive oxygen and nitrogen species; neuroprotective molecules such as neuroprotection D1; as well as local blood flow and genetic modifiers. The complexity of these mechanisms and their involvement in AD pathophysiology have been comprehensively reviewed by Heneka et al. [25], Hampel et al. [44], and Thakur et al. [45] (Fig. 2).

## TNF Functions under Homeostatic Conditions

TNF-α, a key proinflammatory cytokine, shows a reduced level and expression in the adult brain when compared with developmental stages and neuroinflammatory or neurodegenerative disease states in human postmortem tissue and rodent studies [46–49]. It is also secreted by several cell types, including cells of the monocytic lineage, which are the primary precursors of microglia [50]. It also promotes and regulates glutamatergic [51–53] and gamma-aminobutyric acid (GABA) transmission [52, 54, 55] in the CNS, enhancing and reducing the excitatory synaptic and inhibitory transmission, respectively, in hippocampal slices [52] and other neuronal circuits [46]. TNF amplifies excitatory transmission by enhancing the release of glutamate from astrocytes [44, 54]. TNF is reported to play several roles in CNS. It stimulates sleep [56] by activating NF-κB pathways, which are vital for enhancing adenosine A1 receptors [57]. It acts on neurons in sleep regulatory areas, like the basal forebrain and hypothalamic preoptic area, in the brain, thereby enhancing sleep [58]. In mice, the i.p. injection of TNF induces a significant increase in non-rapid eye movement sleep duration and a decrease in slow-wave activity [59].

Growing evidence highlights the critical role of both innate and adaptive immune processes in AD pathology. In the healthy adult brain, TNF is expressed at low, constitutive levels [46, 47]. Among different cellular sources, microglia are considered the main producers [50]. TNF-α influences numerous neuronal activities, including glutamatergic and GABAergic transmission, thereby shifting the balance toward excitatory synaptic signaling in the hippocampus and potentially in other neuronal circuits [52]. This excitatory effect is further strengthened by TNF-α–induced glutamate release from astrocytes [46, 60]. Beyond synaptic modulation.

## TNF and its Receptors in AD

Nevertheless, TNF-α also amplifies inflammatory cascades by inducing other cytokines such as IL-1, IL-6, and IL-8, sustaining chronic neuroinflammation if not counterbalanced by anti-inflammatory mediators like IL-10 [61]. Importantly, TNF-α can promote amyloid pathology by upregulating APP and BACE1 expression, enhancing γ-secretase activity, and increasing Aβ production [62, 63]. Sustained TNF-α signaling may also impair microglial clearance of Aβ, reinforcing a self-perpetuating inflammatory cycle. Although less well studied, emerging evidence suggests that TNF-α may also contribute to tau hyperphosphorylation [64]. While microglial activation and gliosis were once considered secondary to neuronal loss in AD, genetic studies now suggest that immune-related pathways, including those mediated by microglia and astrocytes, are active early in disease progression [65, 66]. Of particular interest is TREM2, an innate immune receptor expressed on microglia, for which mutations have been linked to an increased risk of AD [67]. TREM2 deficiency is believed to disrupt the regulation of inflammatory cytokines, including TNF-α, via Toll-like receptor pathways. Dysfunctional TREM2 signaling in myeloid cells may therefore enhance systemic TNF-α production and exacerbate AD pathology [68]. In addition, genetic variations within the TNF-α gene itself may influence disease susceptibility [69]. For example, the G308A polymorphism has been associated with elevated TNF-α mRNA and protein expression; however, meta-analyses reveal regional differences in its association with AD risk, underscoring the need for further investigation [70].

### TNFR

The effects of TNF-α in the CNS are mediated through its two receptors, TNF receptor 1 (TNFR1) and TNF receptor 2 (TNFR2), which have distinct but complementary roles [71].

### TNFR1

TNFR1 has been associated with neuroprotection under certain conditions by preventing necrosis; however, its protective capacity in acute CNS injury is limited [46]. Instead of its anti-apoptotic role, it partially explores its neuroprotective action in acute CNS injuries. Neuroprotection through TNFR1 appears to involve both the induction of survival-related genes and caspase-dependent apoptosis when survival cues are insufficient [46]. Mostly, TNFR1 signaling and the FADD/caspase-8 apoptotic pathway induce apoptosis rather than inflammation-mediated necrosis. The neuroprotection facilitated by TNFR1 may operate either by gene induction or by de novo production of survival proteins during the absence of caspase 8-dependent apoptotic signals [46]. Remarkably, TNFR1 flops to protect the oligodendrocytes from immune-mediated damage in multiple sclerosis and experimental autoimmune encephalomyelitis, and neurons from retinal ischemia–reperfusion injury [46]. TNF toxicity in mice was aggravated by the inhibition of pan-caspase, through enhancing oxidative imbalance and mitochondrial dysfunction [72]. Interestingly, blocking caspases exacerbates TNF-induced toxicity, suggesting that TNFR1 signaling can toggle between apoptosis and necroptosis through molecules such as RIP1 and RIP3. Thus, caspase-8–mediated apoptosis may serve as a safeguard against necroptotic cell death, which is more inflammatory [73, 74].

### TNFR2

**Fig. 3:**
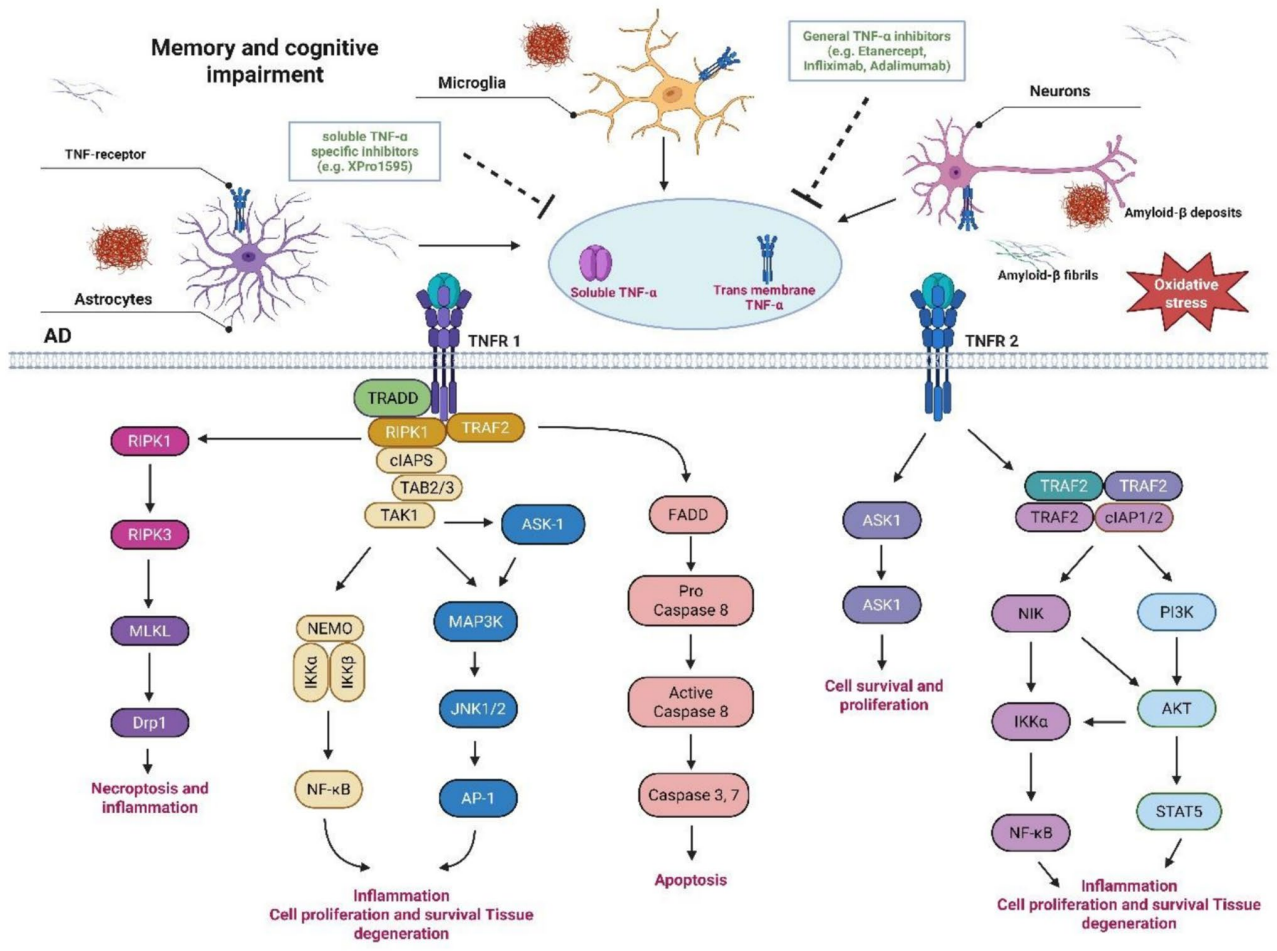
Targeting TNF-α signaling with TNF-α inhibitors in AD pathology. TNF-α plays a central role in AD–associated neuroinflammation by disrupting BBB integrity and activating receptor-mediated inflammatory signaling within the brain. TNF-α enters the brain via BBB transcytosis, increasing barrier permeability and enabling greater peripheral Aβ influx and parenchymal amyloid deposition, which further amplifies local inflammatory burden. In the brain, TNF-α binds to tumor necrosis factor receptors TNFR1 and TNFR2 on resident cells, triggering intracellular signaling cascades that reinforce TNF-α production through positive feedback mechanisms. TNFR1 interacts with both transmembrane TNF-α (tm-TNF-α) and its soluble form generated by TACE/ADAM17 cleavage, whereas TNFR2 preferentially binds tm-TNF-α with higher affinity. TNFR1 activation can initiate apoptotic and necroptotic pathways via death-domain interactions with the adaptor TRADD, whereas both TNFR1 and TNFR2 recruit cIAP1/2 to activate canonical NF-κB signaling. Additionally, TNFR2 weakly engages TRAF2 to stimulate the alternative NF-κB pathway. The cytosolic availability of cIAP1/2 and TRAF2 critically determines signaling outcomes in cells expressing both receptors, as their depletion following TNFR2 activation can bias TNFR1 signaling toward cell death rather than survival. Collectively, these interconnected processes establish a self-perpetuating neuroinflammatory cycle that accelerates neurodegeneration, suggesting that targeting membrane-bound and/or soluble TNF-α may be an effective early therapeutic strategy to interrupt AD-related inflammatory pathology and promote neuroprotection. Created with BioRender.com

TNFR2, by contrast, is strongly linked to neuroprotection and repair. It is expressed on regulatory T cells, endothelial cells, oligodendrocyte lineage cells, and select neurons and can induce pro-survival signaling by procuring TRAF2 [28] and activating the PI3K/NF-κB pathway [75–78], thereby protecting against excitotoxic and ischemic damage [75, 76] (Fig. 3). TNFR1 and TNFR2 act synergistically to enhance survival signaling, with TNFR2 augmenting TNFR1’s protective and apoptotic responses [79]. Additionally, TNFR2 supports the proliferation of oligodendrocyte precursors and remyelination, thereby reinforcing its role in CNS repair [80]. Furthermore, TNFR2 activation is linked to the stimulation of Akt/protein kinase B, which is essential for neuroprotection against ischemia–reperfusion injury and excitotoxicity [46]. TNFR2 may promote repair mechanisms by inducing proliferation and remyelination of oligodendrocyte precursor cells. [79, 80]. TNF is reported to induce the production of both pro-inflammatory cytokines (IL-1, IL-6, and IL-8, which are involved in the development of chronic inflammation) and anti-inflammatory cytokines (IL-10) [81].

The stimulation of TNFR2 with the administration (implantation of osmotic pumps, or systemically by intraperitoneal injections) of its agonist (NewStar2) in a transgenic Aβ-overexpressing mouse model of AD attenuated Aβ deposition, cognitive impairments and reduced phagocytic activity of glial cells in the CNS [82]. Almazan et al., [83] indicated that the intrahippocampal administration of a TNFR2-specific blocking antibody improved recognition memory, spatial memory, and synaptic potentiation in response to chemically induced LTP (cLTP) in 5xFAD mice. Moreover the lack of TNFR2 (selective ablation of TNFR2) in astrocytes resulted in changed expression of key proteins involved in hippocampal synaptic transmission and plasticity, increased hippocampal gliosis, impaired LTP, and deficits in learning and memory in males as compared to females [84]. Taken together, these findings highlight the pivotal role of TNF-α in AD pathophysiology, acting at the intersection of neuroinflammation, synaptic regulation, sleep, and neurodegeneration. Ongoing studies in both human and animal models continue to clarify its dual protective and pathological roles, while future research will determine whether modulating TNF-α or its receptors represents a viable therapeutic strategy in AD.

In AD, TNFR1 signaling plays a main role in neurodegenerative processes particularly from earliest stages of the disease. The chronic TNFR1 activation by the soluble form of TNF-α triggers amyloid genesis process by enhancing the protein expression and activity of β-secretase through the NF-κB pathway, resulting in increased Aβ production and plaque formation [85]. TNFR1 is the key initiator of neuronal necroptosis, a form of inflammatory programmed cell death; research on post-mortem AD brains has identified an activated RIPK1/RIPK3/MLKL "necrosome" complex in hippocampal neurons, which correlates inversely with neuron density. This receptor also contributes to auto-lysosomal dysfunction by impairing lysosomal acidification through the downregulation of V-ATPase subunits, which prevents the effective degradation of toxic cellular cargo and damaged mitochondria [86]. Furthermore, TNFR1 signaling disrupts the blood-CSF barrier in the choroid plexus, leading to morphological damage of epithelial cells and reduced clearance of Aβ [85]. Consequently, selective TNFR1 antagonists are being investigated as potential therapies to halt AD progression while preserving the homeostatic benefits of TNFR2 signaling [87]. Although both TNFR1 and TNFR2 can intermediate neuroprotective effects under specific conditions, most of the evidence indicates that TNFR1 signalling is primarily associated with neurodegenerative and pro-inflammatory outcomes, but TNFR2 signalling more consistently promotes neuroprotection and tissue repair.

## TNFR1 and TNFR2 Signaling Pathway

TNF is a key cytokine, first formed as a transmembrane protein (tmTNF) that binds to TNF receptor 2 (TNFR2: CD120b or p75/p80). If it is sliced from the membrane, TNF becomes a soluble form (solTNF) that mainly binds to TNF receptor 1 (TNFR1: CD120a or p55/p60) [88]. Both TNFR1 and TNFR2 activate a few common signaling pathways [89]. TNFR2 activation induces positive effects such as increased cell viability, promotion of neurogenesis, and induction of autoimmunity [17–19], whereas TNFR2 activation mostly induces destructive effects such as induction of apoptosis, reduction in neuronal plasticity, and aggravation of inflammation [90–92].

The binding of solTNF to the cognate receptor results in the procurement of the TNF adaptor protein (TNF receptor-associated death domain -TRADD), which leads to the binding of other cytosolic adaptor proteins like FAS-associated death domain (FADD), TNF receptor-associated factor 2 (TRAF2), and receptor-interacting protein (RIP). TRAF2 activates the mitogen-activated protein kinase (MAPK) pathway, leading to c-Jun N-terminal kinase (JNK) activation and enhanced transcriptional activity (Fig. 3). The RIP is a protein kinase that triggers NF-κB activation by phosphorylating IκB kinase (IKK). On the other hand, the FADD pathway activates caspase 8, thus inducing a caspase-mediated apoptosis [93, 94]. TNF-mediated p38 MAPK pathway activation has also been involved in the induction of IL-6 production [95].

TNFR2 activation leads to enlistment of TRAF2 [96], cIAP1/cIAP2 [97], and HOIP, a LUBAC component [98], which shapes the TNFR2 signaling complex. cIAP-induced K63-mediated polyubiquitination of the SC is essential for the procurement of HOIP that enhances M1-ubiquitination [98]. The HOIP and cIAP1 are needed for TNFR2-mediated canonical NF-κB activation [98, 99]. TNFR2 was reported to induce the non-canonical NF-κB pathway, also [100]. TRAF2 is degraded by receptor internalization and lysosomal degradation [101]. This leads to the accumulation of the kinase NIK, which results in the phosphorylation and activation of IKKα. This leads to the conversion of the p100 subunit of NF-κB to p52 and the enhanced nuclear translocation of p52/RelB NF-κB heterodimers [102] (Fig. 3).

The activation of both TNFR1 and TNFR2 may induce c-Jun N-terminal kinase (JNK) [103] and the p38 MAPK pathways [104, 105]. Interestingly, mitochondrial aminopeptidase P3 (APP3) was found as a new constituent of the TNFR2 signal complex that modulates TNFR2-mediated JNK phosphorylation [105]. Moreover, TNFR2 activation increases human regulatory T cells (Tregs) proliferation by the non-canonical NF-κB pathway in [106]. In the mouse, Tregs mediated activation of p38 and MAPK signalling pathways was a key process in TNFR2-induced proliferation [104] (Fig. 3). TNFR2 activation induces phosphatidylinositol 3-kinase (PI3K)-mediated protein kinase PKB/Akt phosphorylation by an unknown mechanism [107, 108]. The D3 OH group of the inositol ring of the phosphatidylinositol-4,5-bisphosphate (PIP2), a plasma membrane lipid molecule, is phosphorylated by PI3K, leading to the formation of phosphatidylinositol 3,4,5-bisphosphate (PIP3), a second messenger [109]. PKB/Akt is then recruited to the membrane by its binding of pleckstrin-homology (PH) domains to PIP3 [110]. Then, a conformational alteration occurred in the structure of PKB/Akt, which gets phosphorylated at threonine 308 and serine 473 by PDK-1 [111] and Rictor/mammalian target of rapamycin (mTOR) complex [112], respectively. Activated PKB/Akt enhances cell viability and proliferation [113, 114].

## Role of TNF Receptor Modulators in AD

In AD research, targeting TNF receptors involves using TNFR1 antagonists (to block harmful inflammation) and TNFR2 agonists (to promote neuroprotection and regeneration). This approach aims to rebalance the body's inflammatory response, which is dysregulated in AD [115].

Both clinical studies and animal model experiments fail to identify the specific roles of TNFR1 and TNFR2 in AD. Although TNFR2 activation exerts neuroprotective effects in various disease models, its impact on Aβ-mediated neuropathology and cognitive dysfunction remains to be elucidated. Jiang et al. [115] demonstrated that the genetic deletion of *TNFRII* in AD transgenic mouse model novel showed AD-like pathology, i.e. plaque formation (elevated Aβ levels and BACE activity) and microglial activation, occurs as early as 6 months of age, which is reversed in overexpressed transgenic mice.

Therefore, this review discusses how selective inhibition of TNFR1 can revoke Aβ- and Tau-mediated neuropathology in AD. Previous studies have shown that pre-plaque-associated neuropathology in AD mice can be prevented by inhibiting the sol-TNF signaling pathway [116]. Moreover, genetic ablation of TNF reduces plaque formation by reducing Aβ synthesis in AD mice [117]. The genetic deletion of TNFR-1 or the administration of TNF regulators or inhibitors reduced Aβ toxicity in AD mice [116, 118–121]. These experiments demonstrated that inhibiting TNF signaling reverses the toxic effect of amyloid in AD pathology. This review discusses the development of anti-TNF therapy for AD and describes its prospects. Currently, there are five approved anti-TNFs: 1) Etanercept, a dimeric fusion of TNFR2, with Fc regions of IgG1 (Enbrel®); 2) Infliximab, an IgG anti-human chimeric monoclonal antibody (Remicade®); 3) Adalimumab, a humanized monoclonal antibody (Humira®); 4) Golimumab, a humanized antibody(Simponi®); and 5) Certolizumab, a PEGylated Fab region of antibody (Cimzia®). However, many more ‘biosimilars’ are on the way to market, as many are in development. This review proposes summarizing the key functions of TNF inhibitors and the role of TNF in AD and identifying a potential target for AD in future preclinical and clinical studies. The summary of the advantages/disadvantages of TNF receptor inhibitors with principal features relevant to AD are listed in Table 1.

**Table 1:** Summarizing the advantages/disadvantages of TNF receptor inhibitors with principal features relevant to AD

TNF receptor inhibitors/agonists	Advantages	Disadvantages	Target	CNS penetration	Safety	Clinical status	Future directions	References
Etanercept	Effectively reduced systemic inflammation Long-term use is approved and showed a reduction in AD risk;	It blocks the harmful TNFR1-mediated signaling and the beneficial TNFR2-mediated signaling	TNFR1 and TNFR2 (Higher affinity for solTNF compared to tmTNF)	Partial	Increased risk of serious infections and risk of certain malignancies	Small human pilot studies and large observational studies were carried out, but large-scale randomized controlled trials in AD patients are needed	Conduct well-designed, large-scale randomized controlled trials in patients; optimize CNS delivery strategies; investigate TNFR1-selective variants to preserve TNFR2-mediated neuroprotective signaling	[122–128]
Infliximab	Reduced AD Risk and inflammation	Preclinical Efficacy	TNFR1 and TNFR2 (Binds both solTNF and tmTNF)	does not readily cross	Tuberculosis reactivation and fungal infections, malignancies and moderate-to-severe heart failure	one case report described cognitive improvement with intrathecal administration	Modified formulations or delivery methods to enhance BBB penetration; controlled clinical studies needed to validate cognitive benefits observed in case reports; assess combination therapy with AD-specific disease-modifying agents	[129, 130]
Adalimumab	reducing the inflammatory cascade and potentially decreasing Aβ plaque formation by downregulating BACE1 expression	Blocks neuroprotective TNFR2 signaling Risk of demyelination Poor CNS penetration	It binds directly to the TNF-alpha ligand and blocks the cytokine from interacting with both TNFR1 and TNFR2 (Neutralizes both soluble and membrane-bound forms of TNF)	Cross only after perispinal injection	Well-established safety from years of use in other conditions	retrospective human studies	Investigate peri spinal delivery for CNS targeting; evaluate long-term cognitive outcomes in future AD cohorts; study interactive effects with anti-amyloid or anti-tau therapies	[122, 127, 131, 132]
XPro1595	Reduces Neuroinflammation Targets inflammation without immunosuppression	Not yet approved Target is narrow	Selectively neutralizes sTNF and does not inhibit mTNF	Designed for Blood Brain Barrier penetration and reach the CNS	Limited safety data	Phase 2 clinical development for AD	Advance Phase 2 and Phase 3 clinical trials to confirm efficacy and safety in AD; validate biomarkers of solTNF inhibition; search earlier intervention stages such as mild cognitive impairment	[133–136]
Golimumab	Highly effective for approved inflammatory conditions	No evidence of efficacy for AD due to limited CNS penetration	targets and blocks TNF-alpha (Binds both solTNF and tmTNF)	Does not cross	Black box warning for serious infections and potential malignancy Risk of lupus-like syndrome, heart failure exacerbation, and demyelinating disorders	no published clinical trials or approved indications	Investigate structural adjustments or another delivery platforms to improve CNS penetration; conduct preclinical AD-specific studies before considering clinical trials	[137, 138]
Certolizumab	Neutralizes peripheral TNF-alpha, which is implicated in driving central nervous system (CNS) inflammation and potentially contributing to AD progression	Does not directly target central nervous system (CNS) specific pathologies like Aβ plaques or tau tangles, which are core features of AD pathology	Peripheral TNF-alpha (Binds both solTNF and tmTNF)	Limited BBB penetration	Extensive long-term safety data from non-AD conditions	Current clinical evidence is limited to small or ongoing investigational studies;	Evaluate indirect effects of peripheral TNF-α neutralization on CNS inflammation using biomarker-driven studies; explore novel CNS-targeted formulations; focus on mechanistic studies relating systemic TNF reduction to AD progression	[139]
Suramin	Broad anti-inflammatory and neuroprotective mechanisms and potential effects on AD biomarkers (tau, Aβ aggregation)	Non-selective action on many receptors and enzymes can lead to off-target effects and complex toxicities	(P2 antagonism, cGAS-STING inhibition)	CNS toxicity is not a significant issue as it does not typically cross the BBB	Long clinical history provides extensive safety data; low doses in pilot studies (for ASD) were well-tolerated	research is currently in the preclinical stage. Studies have primarily been in cell cultures and animal models showing promising neuroprotective and cognitive-enhancing effects by targeting inflammatory pathways	Develop more selective analogs with reduced off-target toxicity; expand animal studies focused on cognition and AD pathology; assess low-dose regimens and combination strategies targeting innate immune pathways	[140, 141]
SPD304	Inhibiting excessive TNF-α activity could reduce neurotoxicity and protect neurons	SPD304 itself is not suitable for a drug optimization program due to its poor physicochemical and pharmacological properties	promoting the dissociation of the TNF-α trimer, thus blocking the interaction of TNF-α with its receptors (TNFR1 and TNFR2)	As a small molecule, it theoretically has a higher potential for blood–brain barrier (BBB) penetration compared to large biologic inhibitors (e.g., antibodies)	Selective targeting harmful TNF signaling could offer neuroprotective effects	SPD304 is not a drug; it is an early-stage research compound and has no clinical use	Use SPD304 for medicinal chemistry optimization; Develop derivatives with improved pharmacokinetics and safety; validate TNF-α trimer disruption as a viable therapeutic mechanism in AD models	[142–144]
Physcion-8-O-β-d-monoglucoside	natural anthraquinone with potential neuroprotective properties and ameliorative effects against dementia in in vitro and animal studies	Lack of human data: Efficacy and safety are primarily from in vitro and animal studies	inhibit TNF-α-induced cytotoxicity and apoptosis via TNF-R1 in cell studies	cross the blood–brain barrier	Natural compound source: Derived from natural products, potentially offering a better safety profile than some synthetics	primarily in pre-clinical research status for AD	Perform pharmacokinetic and toxicity profiling; validate efficacy in advanced AD animal models; progress toward standardized formulations and early-phase clinical feasibility studies	[145–147]

### Etanercept

Etanercept (Enbrel) is a fusion protein synthesized by rDNA technology and a potent inhibitor of TNF(acts as a soluble TNF receptor), which binds to TNF-alpha and TNF-beta [122]. Etanercept comprises two p75 TNF receptors linked to the human IgG (Fc portion) [122]. Etanercept blocks the effects of TNF, which is enhanced in rheumatoid arthritis, psoriasis, juvenile idiopathic arthritis, psoriatic arthritis, and ankylosing spondylitis. Etanercept has also been shown to reduce motor and cognitive disorders, cerebral edema, and other markers of neuronal injury, including the activation of astrocytes and microglial cells, as well as the modulation of other inflammatory intermediates. Thus, this molecule can enter the brain tissue and be shown to reduce the cognitive and motor deficits by enhancing neurogenesis.

#### Mechanism of Action of Etanercept

Etanercept is a large drug molecule encompassing 14 O-glycans, 6 N-glycans, and 29 disulfide bonds [148, 149]. In white blood cells, lymphocytes and macrophages can synthesize TNF. All nucleated cells contain TNF receptors on their surface. The immune response was enhanced by the extravasation of numerous white blood cells to inflammatory sites, and this process was further exacerbated by some extracellular mechanisms that increased inflammation. Etanercept reduces the inflammatory response by inhibiting TNF, which is mainly useful for the treatment of autoimmune diseases. This drug reduces the efficacy of the natural TNF molecule by acting as a decoy receptor that binds to TNF [150], and dull the immune response. Although this drug mimics the inhibitory effects of natural soluble TNF receptors, the main difference lies in the enhanced half-life in the bloodstream. Therefore, this molecule extends a prolonged and long-lasting biological efficacy than its natural counterpart [151].

Li and colleagues [152–154] Recently, it has been demonstrated that intravenous administration of etanercept to transgenic AD mice (3xTg) significantly enhances spatial, long-term, and working memory, as assessed by behavioral tests (Morris water and Y-maze tasks). Furthermore, this drug reduces the neuronal injury and cytokine levels in a transgenic mouse model of AD. It also efficiently reduced the activation of JNK and NF-κB signaling pathways, which are the key pathways of neuroinflammation in animal models [152, 154] (Fig. 3). Experiments carried out in the amyloid-induced AD mouse model have indicated that there is a reduced level of TNF in the brain and an enhancement in cognitive function [64]. It is an anti-TNF drug effective in reducing cognitive defects in AD patients [45, 155]. The synthesis of ROS was normally enhanced by TNF through the activation of NADPH oxidase [156] (Fig. 3). Additionally, this molecule was found to diminish the oxidative stress as seen by reduced malondialdehyde levels and enhanced activities of superoxide dismutase and glutathione peroxidase [156].

Etanercept significantly downregulated the hyperphosphorylation of tau proteins and microgliosis in the brains of male and female PS19 mice (a transgenic AD mouse model). Treatment with TfRMAb-TNFR and etanercept enhanced neuronal activity by significantly increasing PSD95 expression and ameliorating hippocampal neurodegeneration in the transgenic mice. Chronic etanercept administration downregulated locomotory hyperactivity in male transgenic mice (Fig. 3). The blood parameters of etanercept-treated animals remain largely unchanged, except for a significant enhancement in platelet levels [64]. A six-month open-label pilot study suggested that inhibiting TNF-alpha may be a promising approach for treatment and recommended the need for future randomized and placebo-controlled clinical studies [123]. The main disadvantage of using etanercept as an anti-AD drug is its larger size, which hinders its entry into the blood–brain barrier. This mainly affects the usage of etanercept as a potent anti-inflammatory agent.

### Infliximab

Infliximab (Remicade, Inflectra, Renflexis, infliximab-dyyb, and infliximab-abda) is a chimeric monoclonal antibody, used in the treatment of various autoimmune disorders like Crohn's disease, rheumatoid arthritis, ulcerative colitis, psoriasis, ankylosing spondylitis, psoriatic arthritis, and Behçet's disease. It is normally injected intravenously at intervals of 6 to 8 weeks [157]. Currently, two biosimilars of this drug are available on the market. Inflectra (the first biosimilar drug) was approved by the FDA in 2016, and Ixifi (the second biosimilar drug) in 2017. The average half-life of this drug is 7.7 to 9.5 days. Infliximab is a κ-κ-chain-containing monoclonal IgG1 antibody with high affinity for both the transmembrane and soluble forms of TNF, thereby interrupting pro-inflammatory signaling pathways. The interaction between TNF and its receptors is prevented by binding TNF with the antibody. Further, inhibited actions of TNF result in the downregulated production of pro-inflammatory cytokines like IL-1and IL-6, decreased migration of leukocytes and lymphocytes to the inflammatory sites, initiation of apoptotic cascade in TNF-producing cells, including activated T cells and monocytes, enhanced concentration of NF-κB inhibitor, and downregulated levels of acute phase proteins and endothelial adhesion molecules [158]. These inhibitory actions on TNF were observed in human endothelial cells, fibroblasts, B and T cells, neutrophils, and epithelial cells. Infliximab also worsens the synthesis of tissue-degrading enzymes in chondrocytes and/or synoviocytes.

#### Mechanism of Action of Infliximab

Infliximab contains both murine and human components (chimeric protein), synthesized from rDNA technology, and mainly used to inhibit TNF [159]. The intracerebroventricular injection of infliximab (150 μg for 3 days) to APP/PS1 mice (transgenic mouse model) of AD was compared to controls administered with IgG via the same route and schedule [120]. Aβ deposits decreased by 40–60%, and tau accumulation was reduced by 70% after 3 days of infliximab treatment in the mice. Similarly, the levels of TNF in the brain were significantly reduced by days 3 and 7, and by day 14 (11 days after the end of the treatment), the TNF levels returned to baseline [120] (Fig. 3). A case report by Shi et al. [160] reported that intrathecal infliximab enhances cognition and provides a higher level of therapeutic approach against TNF-involved pathogenesis of AD and other possible neurodegenerative disorders, such as Parkinson's disease and amyotrophic lateral sclerosis.

Visual recognition memory impairment and perturbed muscarinic acetylcholine receptor–dependent long-term depression (mAChR-LTD) were induced in mouse perirhinal cortex (PRh) slices by Aβ oligomers. Infliximab treatment enhances the visual recognition memory impairment and clears the Aβ effect on mAChR-LTD in mice [161]. Mohamad et al. [162] reported that Infliximab treatment in doxorubicin-administered rats remarkably reverses damaging effects such as AD-like brain injury, upregulated amyloid burden, increased neuroinflammation and apoptosis, and multifocal histological injury in the cerebral cortex with widespread vacuolations (Fig. 3).

### Adalimumab

Adalimumab (Humira) is a biological, fully human, recombinant immunoglobulin G (IgG) anti-TNF monoclonal antibody (mAb) with high affinity, used for the treatment of numerous autoimmune and inflammatory disease conditions, including rheumatoid arthritis, ankylosing spondylitis, psoriatic arthritis, Crohn's disease, and ulcerative colitis. Adalimumab (1330 amino acids) is working as a TNF inhibitor and has a molecular weight of ~ 148 kDa [163]. Adalimumab inhibits both the soluble and transmembrane forms of TNF binding to TNFR1 and TNFR2 receptors. Adalimumab disrupts the cytokine-mediated inflammatory process by neutralizing the binding of the soluble TNF and transmembrane TNF to TNFR1 and TNFR2 receptors. Adalimumab has increased specificity to TNF and low immunogenic properties due to the similar structure and function of human IgG1 [164, 165]. Adalimumab is also available in a biosimilar form under the brand names Amgevita, Hyrimoz, Idacio, Imraldi, and Yuflyma. These biosimilars also inhibit the immune system and reduce inflammation, just as adalimumab does.

#### Mechanism of Action of Adalimumab

Adalimumab specifically inhibits the TNF receptors without interacting with or binding to other cytokines, such as lymphotoxin or interleukins. The capability of adalimumab in arthritis can be assessed by its osteogenic hallmark properties. TNF inhibition inactivates the NF-κB ligand and receptor in stromal or osteoblast cells, thereby reducing cartilage and bone loss. In arthritis serum, matrix metalloproteinases 1 and 3 (MMP-1 and MMP-3) were downregulated by TNF inhibitors. These desirable properties of TNF inhibitors contribute to the treatment of arthritis [164]. Biological reactions such as elevation of adhesion molecules (ELAM-1, VCAM-1, and ICAM-1) during in progression of inflammation are improved by adalimumab [166].

Adalimumab treatment significantly reduces neuropathy, neuroinflammation, and BDNF expression in Aβ1-40-induced AD mice. The mechanism of action of adalimumab involves the activation of the NF-κB signaling pathway. TNF inhibitor adalimumab reduces TNF-activated phosphorylation of serine 536 residues in NF-κB p65, with degradation of inhibitor of κB (IκB, and the levels of TNF and interleukin-6 (IL6) expression in Aβ1-40 induced AD mice [167]. These outcomes suggested that cognition, neuroprotection, and neuroinflammation were improved by the adalimumab treatment in Aβ1-40-induced AD mice. In another study, adalimumab treatment improves memory, reduces neurol loss in the hippocampus, microglial activation, and M1/M2 polarization in vascular dementia rats. Adalimumab treatment particularly reduces NF-κB activity and lowers oxidative stress levels in rats with vascular dementia. These findings suggested that adalimumab may be used for the treatment of clinical trials (Clinical trial number: not applicable) with Vascular dementia [168] (Fig. 3).

### XPro1595

XPro1595 (Pegipanermin) is a selective TNF inhibitor that neutralizes only soluble TNF. XPro1595 is a trimer of three subunits with chemical and biological functions similar to those of human solTNF. XPro1595 has two amino acid replacements that maintain heterotrimeric activity with the innate TNF and prevent binding to TNF receptors. The combination of XPro1595 and innate TNF monomers lacks the binding affinity with the TNF receptor. At least a tenfold higher concentration of XPro1595 than the innate solTNF can selectively neutralize more than 99% within minutes [169]. This is a unique property of XPro1595 that distinguishes it from other TNF inhibitors. FFDA-approved TNF inhibitors that inhibit both solTNF and tmTNF. XPro1595 is not affecting the tmTNF activity in the immune response against infections [150]. There is no evidence of known side effects of XPro1595 treatment in various pre-clinical inflammatory disease models [133, 170–173]. XPro1595 treatment is safe and well-tolerated in cancer patients in clinical trials (Clinical trial number: not applicable) [174]. The second trial study shows the ability to reduce neuroinflammation in the brains of AD patients [133, 175, 176].

#### Mechanism of Action of XPro1595

Three mouse models of AD preclinical studies were clearly defined to evaluate the efficacy of XPro1595. Administration of XPro1595, two subcutaneous injections/week for two months, reduces amyloid protein accumulation in the brain, immune cell infiltration, and enhances the synaptic plasticity [133, 177]. In TgCRND8 mice, subcutaneous injection of XPro1595 for 30 days after 6 months resulted in reduced amyloid accumulation and improved synaptic function compared with untreated mice [173]. 3xTg mice showed reduced pre-plaque amyloid pathology after intracranially administration of XPro1595 [116]. Intracranial administration of XPro1595 reduces microglial activation and enhances synaptic plasticity and cognition in old rats [170]. Inhibiting solTNF with XPro1595 was reported to attenuate the AD risk associated with the effects of a high-fat, high-glucose diet on insulin metabolism and immune and neural function [178, 179]. XPro1595 treatments in other neurological models of Parkinson’s disease and Huntington’s disease have shown improved outcomes in pre-clinical studies; there are ongoing clinical trials (Clinical trial number: not applicable) [134, 180, 181]. In company-sponsored studies, XPro1595 reduced some markers of inflammation but did not prevent dopaminergic neuron degeneration in rhesus monkeys with progressive Parkinsonism [182] (Fig. 3).

## Other TNF Inhibitors in Chronic Inflammatory Conditions

Non-selective TNF inhibitors like etanercept, infliximab, and adalimumab are large molecules that generally do not cross the intact blood–brain barrier (BBB) but are still relevant to AD treatment due to the link between systemic and neuroinflammation. So, the role of TNF-α inhibitors are discussed in the following sections.

### Golimumab

Golimumab is a fully human IgG1 monoclonal antibody developed using a murine hybridoma cell line. The hybridoma was generated from splenocytes of Medarex UltiMAb® (Medarex, Princeton, NJ, USA) transgenic mice engineered to produce human antibodies. Golimumab was the first anti-TNFα therapy approved for once-monthly subcutaneous administration and has been approved in both the United States and Europe for the treatment of moderate to severe rheumatoid arthritis, psoriatic arthritis, and ankylosing spondylitis.

Pharmacokinetic studies indicate that a single 100 mg intravenous dose of golimumab yields a higher maximum serum concentration (Cmax: 29.5 μg/mL) compared to the same dose administered subcutaneously (Cmax: 6.3 μg/mL), although the terminal half-life is similar between routes (IV: 11.8 days; SC: 10.9 days) [183]. Following subcutaneous injection, peak serum levels are typically reached approximately 3.5 days later. Co-administration with methotrexate has been shown to prolong the drug’s half-life and elevate steady-state trough concentrations [184], likely by reducing the formation of anti-drug antibodies (ADAs) that promote faster clearance. In patients with detectable ADAs, the half-life is shortened to approximately 2.9 days. Clinical outcomes from the Phase III GO-FORWARD trial demonstrated that monthly treatment with 100 mg golimumab, in combination with methotrexate, achieved an ACR20 response rate of 56.2% at week 14 [185].

### Certolizumab

Certolizumab is a recombinant Fab fragment of a humanized anti-TNF-α antibody conjugated to a 40-kDa polyethylene glycol (PEG) molecule. Unlike most other TNFα inhibitors, which are produced in mammalian cell systems, certolizumab is manufactured in bacteria, potentially lowering production costs. PEGylation, the process of attaching PEG to proteins, is widely used to extend the half-life of smaller biologics by slowing renal clearance, protecting against proteolysis, and reducing immunogenicity. Through this modification, certolizumab achieves a half-life comparable to that of full-length monoclonal antibodies (approximately 13 days) [186].

Most currently available TNFα inhibitors are either complete antibodies or Fc-fusion proteins that contain the Fc fragment, enabling complement activation and antibody-dependent cell-mediated cytotoxicity. Certolizumab, however, lacks the Fc region and therefore does not trigger these immune effector functions. It can be administered either alone or in combination with methotrexate (MTX). In rheumatoid arthritis (RA) monotherapy trials, patients receiving 400 mg certolizumab every 4 weeks achieved an ACR20 response rate of 45.5% compared to 9.3% in the placebo group [187]. When combined with MTX, response rates were higher, with 58.8%–60.8% of patients achieving ACR20 at week 24, compared to 13.6% with placebo [188].

The long-term safety and efficacy of certolizumab in combination with MTX have been evaluated in RA patients. Following 6 months of treatment with 400 mg every 2 weeks, the dose was reduced to 200 mg every 2 weeks for maintenance and continued for up to 5 years. Patients who initially responded to certolizumab maintained clinical improvements through week 232. Among those who had previously failed to achieve ACR20 with other therapies, mean ACR20/50/70 response rates with certolizumab were 68.4%, 47.1%, and 25.1%, respectively [189]. Overall, certolizumab demonstrated durable clinical benefits and was well-tolerated during long-term therapy.

## Small Molecule TNFα Inhibitors

### Suramin

Suramin, a polysulfonated urea derivative, has traditionally been used in the treatment of trypanosomiasis (caused by trypanosome parasites) and onchocerciasis (river blindness due to Onchocerca volvulus infection). Mechanistic studies revealed that suramin disrupts TNFα trimer formation, a critical step required for the generation of its active ligand capable of binding TNFα receptors [190]. Structural analogs of suramin, such as Evans blue and trypan blue, also share the molecular features necessary for TNFα interaction. Both compounds were shown to inhibit the binding of TNFα to TNFR1, with IC50 values of 0.75 mM and 1.00 mM, respectively, compared with suramin’s IC50 of 0.65 mM [140]. Beyond TNFα, suramin was also reported to block CD40 ligand (CD40L) binding to CD40 with high affinity [191]. In vivo, suramin demonstrated therapeutic activity in a collagen-induced arthritis rat model, where daily intraperitoneal administration at 10 mg/kg for 3 weeks reduced inflammation and promoted joint repair [192]. Moreover, it exhibited protective effects in a d-galactosamine (GalN) and lipopolysaccharide (LPS)-induced acute liver injury model [193].

### SPD304

SPD304 is a low-molecular-weight inhibitor of TNFα that disrupts the stability of the TNFα trimer [142]. By binding directly to TNFα, it accelerates trimer dissociation, thereby dismantling the active ligand required for receptor engagement. The compound inhibits TNFα–TNFR1 interaction with an IC50 of 22 μM and suppresses NF-κB activation by 50% at a concentration of 4.6 μM [142]. Beyond TNFα, SPD304 has also been shown to interact with RANKL, another TNF superfamily member, displaying an affinity of 14 μM [194, 195]. In a rat model of ischemia-induced heart failure, the compound demonstrated therapeutic activity [196]. Structural derivatives of SPD304 have been developed in attempts to reduce toxicity; however, none have yet achieved the efficacy and safety profile necessary for advancement into clinical evaluation [197].

### IW927

Carter and colleagues discovered IW927, a small-molecule antagonist of TNFR1, through chemical library screening [198]. This compound effectively inhibited TNFα-TNFR1 interaction (IC50 = 50 nM) and suppressed TNFα-driven IκB phosphorylation (IC50 = 600 nM). Importantly, IW927 showed no detectable binding to TNFR2 or CD40, and cell-based assays suggested a favorable safety profile. A related analog, IV703, was later developed as a “photochemically enhanced” version of IW927. Although IV703 displayed only weak reversible binding to TNFR1, exposure to light enabled covalent attachment to the receptor. Structural studies revealed the crystal complex of IV703 bound to TNFR1, demonstrating that the compound engages a TNFR1 surface site directly involved in TNFα recognition [198].

### Physcion-8-O-β-d-monoglucoside (PMG)

Using surface plasmon resonance (SPR) screening, Cao et al. evaluated extracts from five medicinal herbs and identified Rheum officinale as a source of a TNFR1-binding compound [145]. The active fractions were analyzed by UPLC-QTOF-MS/MS, which revealed a predominant molecule consistent with PMG. Purified PMG was subsequently confirmed to interact with TNFR1 by SPR, showing a binding affinity of 376 nM. Functional studies in L929 cells demonstrated that PMG could counteract TNFα-mediated cytotoxicity, as assessed by cell viability assays and annexin V-FITC/PI double staining. At a concentration of 20 μM, PMG exhibited anti-TNFα activity on par with the known inhibitor SPD304.

## Conclusion

Altogether, preclinical and clinical studies on the role of TNF in AD pathogenesis revealed that TNF affects multiple functions, including neuronal activity, neurotransmission, sleep regulation, and astrocyte-mediated glutamate release. TNF plays a crucial role in chronic inflammation, influences the accumulation of amyloid plaques and the formation of Tau tangles, and ultimately controls disease progression. TNF inhibitors have been continuously associated with reduced risk of AD in epidemiological studies. TNF inhibitors in clinical trials (Clinical trial number: not applicable) studies showing existing outcomes in the development and progression of AD. Interestingly, many studies have reported that various TNF-related molecules are present in preclinical models of AD. These outcomes strongly suggest targeting TNF-mediated disease progression by exploring different inflammatory pathways. There is insufficient clinical data to evaluate the effectiveness of TNF inhibitors in AD. All the existing clinical trials (Clinical trial number: not applicable) are in the initial stages, and the present data are therefore inadequate. In the future, high-quality studies are needed to assess the therapeutic potential of anti-TNF drugs in AD, and this research direction should be pursued promptly.

## Limitations and Future Directions

Most current evidence on selective TNF inhibition comes from animal or in vitro studies, which may not accurately reflect the complexity of human AD pathology. The heterogeneity of Alzheimer’s, including variability in onset, progression, and patient genetics, can significantly influence therapeutic efficacy and limit the generalizability of results. While selective TNF inhibition is designed to reduce neuroinflammation, TNF also plays essential roles in immune regulation, and its long-term suppression could lead to unintended immunological effects. Another major challenge lies in achieving effective CNS concentrations of TNF inhibitors, as many current compounds have limited permeability across the blood–brain barrier. Moreover, there is a lack of long-term data assessing cognitive and neuropathological outcomes following TNF-targeted therapy in Alzheimer’s patients. Future research should focus on developing advanced drug delivery systems, such as nanoparticles, carrier-mediated transport, or intranasal formulations, to enhance CNS bioavailability. Personalized medicine approaches that incorporate genetic, inflammatory, and biomarker profiling could help identify patient subgroups most likely to respond to treatment. Combining TNF inhibition with other therapeutic strategies, such as amyloid- or tau-targeted interventions, may offer synergistic benefits by simultaneously addressing multiple disease mechanisms. Large-scale, multi-year clinical trials are needed to evaluate the long-term safety, tolerability, and efficacy of these approaches in diverse populations. Additionally, mechanistic studies clarifying the specific roles of TNF receptor subtypes (TNFR1 vs. TNFR2) in Alzheimer's-related neuroinflammation could further refine and optimize selective targeting strategies.

## Data Availability

No datasets were generated or analysed during the current study.

## Abbreviations

Aβ: Amyloid-beta
AD: Alzheimer's disease
APOE: Apolipoprotein E
APP3: Aminopeptidase P3
ARIA: Amyloid-related imaging abnormalities
BBB: Blood–brain barrier
CNS: Central nervous system
CSF: Cerebrospinal fluid
FADD: FAS-associated death domain
GABA: Gamma-aminobutyric acid
IFN-γ: Interferon-γ
IKK: IκB kinase
JNK: C-Jun N-terminal kinase
MAPK: Mitogen-activated protein kinase
MRI: Magnetic Resonance Imaging
mAChR-LTD: Muscarinic acetylcholine receptor–dependent long-term depression
PI3K: Phosphatidylinositol 3-kinase
PRh: Perirhinal cortex
PMG: Physcion-8-O-β-d-monoglucoside
RA: Rheumatoid arthritis
RIP: Receptor-interacting protein
solTNF: Soluble tumor necrosis factor
tmTNF: Transmembrane tumor necrosis factor
TNFR1: Tumor necrosis factor receptor 1
TNFR2: Tumor necrosis factor receptor 2
TNF-α: Tumor necrosis factor-α
TRADD: TNF receptor-associated death domain
TRAF2: TNF receptor-associated factor 2
TREM2: Triggering Receptor Expressed on Myeloid Cells 2
WHO: World Health Organization

## References

[1] Global action plan on the public health response to dementia 2017–2025. https://www.who.int/publications/i/item/global-action-plan-on-the-public-health-response-to-dementia-2017---2025. 10.1080/13607863.2018.1544213 30600688

[2] 2023. Alzheimer’s disease facts and figures. *Alzheimer’s & Dementia* 19:1598–1695. 10.1002/alz.13016. 10.1002/alz.13016 36918389

[3] McKhann, Guy M.., David S.. Knopman, Howard Chertkow, Bradley T.. Hyman, Clifford R.. Jack, Claudia H.. Kawas, William E.. Klunk, et al. 2011. The diagnosis of dementia due to Alzheimer’s disease: Recommendations from the National Institute on Aging-Alzheimer’s Association workgroups on diagnostic guidelines for Alzheimer’s disease. *Alzheimer’s & Dementia* 7:263–269. 10.1016/j.jalz.2011.03.005. 10.1016/j.jalz.2011.03.005 PMC3312024 21514250

[4] Masters, Colin L.., Randall Bateman, Kaj Blennow, Christopher C.. Rowe, Reisa A.. Sperling, and Jeffrey L.. Cummings. 2015. Alzheimer’s disease. *Nature Reviews Disease Primers* 1 : 15056. 10.1038/nrdp.2015.56. 27188934 10.1038/nrdp.2015.56

[5] Livingston, Gill, Andrew Sommerlad, Vasiliki Orgeta, Sergi G. Costafreda, Jonathan Huntley, David Ames, Clive Ballard, et al. 2017. Dementia prevention, intervention, and care. *Lancet* 390:2673–2734. 10.1016/S0140-6736(17)31363-6. 28735855 10.1016/S0140-6736(17)31363-6

[6] Guo, Yunliang, Zhongyu Fan, Shuo Zhao, Wei Yu, Xunyao Hou, Shanjing Nie, Song Xu, Cheng Zhao, Junting Han, and Xueping Liu. 2023. Brain-targeted lycopene-loaded microemulsion modulates neuroinflammation, oxidative stress, apoptosis and synaptic plasticity in β-amyloid-induced Alzheimer’s disease mice. *Neurological Research* 45:753–764. 10.1080/01616412.2023.2203615. 37068195 10.1080/01616412.2023.2203615

[7] Dorszewska, Jolanta, Michal Prendecki, Anna Oczkowska, Mateusz Dezor, and Wojciech Kozubski. 2016. Molecular basis of familial and sporadic Alzheimer’s disease. *Current Alzheimer Research* 13:952–963. 10.2174/1567205013666160314150501. 26971934 10.2174/1567205013666160314150501

[8] Reardon, Sara. 2023. Alzheimer’s drug donanemab helps most when taken at earliest disease stage, study finds. *Nature* 619:682–683. 10.1038/d41586-023-02321-1. 37460689 10.1038/d41586-023-02321-1

[9] Christodoulou, Rafail C.., Platon S.. Papageorgiou, Maria Daniela Sarquis, Ludwing Rivera, Celimar Morales Gonzalez, Daniel Eller, Gipsany Rivera, et al. 2025. From lesion to decision: AI for ARIA detection and predictive imaging in Alzheimer’s disease. *Biomedicines* 13 : 2739. 10.3390/biomedicines13112739. 41301832 10.3390/biomedicines13112739 PMC12650076

[10] Sandrone, Stefano, Daniel Moreno-Zambrano, Jonathan Kipnis, and Jan Van Gijn. 2019. A (delayed) history of the brain lymphatic system. *Nature Medicine* 25:538–540. 10.1038/s41591-019-0417-3. 30948855 10.1038/s41591-019-0417-3

[11] Fernández, Jorge A.., Leonel Rojo, Rodrigo O.. Kuljis, and Ricardo B.. Maccioni. 2008. The damage signals hypothesis of Alzheimer’s disease pathogenesis. *Journal of Alzheimer’s Disease* 14:329–333. 10.3233/JAD-2008-14307. 18599959 10.3233/jad-2008-14307

[12] Gilhus, Nils Erik, and Günther. Deuschl. 2019. Neuroinflammation — a common thread in neurological disorders. *Nature Reviews Neurology* 15:429–430. 10.1038/s41582-019-0227-8. 31263256 10.1038/s41582-019-0227-8

[13] Block, Michelle L.., and Jau-Shyong. Hong. 2005. Microglia and inflammation-mediated neurodegeneration: Multiple triggers with a common mechanism. *Progress in Neurobiology* 76:77–98. 10.1016/j.pneurobio.2005.06.004. 16081203 10.1016/j.pneurobio.2005.06.004

[14] Jayaraman, Anusha, Thein Than Htike, Rachel James, Carmen Picon, and Richard Reynolds. 2021. TNF-mediated neuroinflammation is linked to neuronal necroptosis in Alzheimer’s disease hippocampus. *Acta Neuropathologica Communications* 9 : 159. 10.1186/s40478-021-01264-w. 34625123 10.1186/s40478-021-01264-w PMC8501605

[15] Yuan, Junying, Palak Amin, and Dimitry Ofengeim. 2019. Necroptosis and RIPK1-mediated neuroinflammation in CNS diseases. *Nature Reviews Neuroscience* 20:19–33. 10.1038/s41583-018-0093-1. 30467385 10.1038/s41583-018-0093-1 PMC6342007

[16] Montgomery, Sara L.., and William J.. Bowers. 2012. Tumor necrosis factor-alpha and the roles it plays in homeostatic and degenerative processes within the central nervous system. *Journal of Neuroimmune Pharmacology* 7:42–59. 10.1007/s11481-011-9287-2. 21728035 10.1007/s11481-011-9287-2

[17] Tansey, Malu and McAlpine. 2008. Neuroinflammation and tumor necrosis factor signaling in the pathophysiology of Alzheimer&rsquo;s disease. *Journal of Inflammation Research* 29. 10.2147/JIR.S4397. 10.2147/jir.s4397 PMC3218716 22096345

[18] Sala Frigerio, Carlo, Leen Wolfs, Nicola Fattorelli, Nicola Thrupp, Iryna Voytyuk, Inga Schmidt, Renzo Mancuso, et al. 2019. The Major Risk Factors for Alzheimer’s Disease: Age, Sex, and Genes Modulate the Microglia Response to Aβ Plaques. *Cell Reports* 27:1293-1306.e6. 10.1016/j.celrep.2019.03.099. 31018141 10.1016/j.celrep.2019.03.099 PMC7340153

[19] Heppner, Frank L.., Richard M.. Ransohoff, and Burkhard Becher. 2015. Immune attack: The role of inflammation in Alzheimer disease. *Nature Reviews Neuroscience* 16:358–372. 10.1038/nrn3880. 25991443 10.1038/nrn3880

[20] Ising, Christina, Carmen Venegas, Shuangshuang Zhang, Hannah Scheiblich, Susanne V.. Schmidt, Ana Vieira-Saecker, Stephanie Schwartz, et al. 2019. NLRP3 inflammasome activation drives tau pathology. *Nature* 575:669–673. 10.1038/s41586-019-1769-z. 31748742 10.1038/s41586-019-1769-z PMC7324015

[21] Liu, Chang, Guohong Cui, Meiping Zhu, Xiangping Kang, and Haidong Guo. 2014. Neuroinflammation in Alzheimer’s disease: Chemokines produced by astrocytes and chemokine receptors. *International Journal of Clinical and Experimental Pathology* 7:8342–8355. 25674199 PMC4314046

[22] Ma, Yingjuan, Bin Ma, Yuying Shang, Qingqing Yin, Yan Hong, Song Xu, Chao Shen, Xunyao Hou, and Xueping Liu. 2018. Flavonoid-rich ethanol extract from the leaves of *Diospyros kaki* attenuates cognitive deficits, amyloid-beta production, oxidative stress, and neuroinflammation in APP/PS1 transgenic mice. *Brain Research* 1678:85–93. 10.1016/j.brainres.2017.10.001. 29038004 10.1016/j.brainres.2017.10.001

[23] Pascoal, Tharick A.., Andrea L.. Benedet, Nicholas J.. Ashton, Min Su Kang, Joseph Therriault, Mira Chamoun, Melissa Savard, et al. 2021. Microglial activation and tau propagate jointly across Braak stages. *Nature Medicine* 27:1592–1599. 10.1038/s41591-021-01456-w. 34446931 10.1038/s41591-021-01456-w

[24] Holmes, C., C. Cunningham, E. Zotova, D. Culliford, and V. H. Perry. 2011. Proinflammatory cytokines, sickness behavior, and Alzheimer disease. *Neurology* 77:212–218. 10.1212/WNL.0b013e318225ae07. 21753171 10.1212/WNL.0b013e318225ae07 PMC3136056

[25] Heneka, Michael T., Monica J. Carson, Joseph El Khoury, Gary E. Landreth, Frederic Brosseron, Douglas L. Feinstein, Andreas H. Jacobs, et al. 2015. Neuroinflammation in Alzheimer’s disease. *The Lancet Neurology* 14:388–405. 10.1016/S1474-4422(15)70016-5. 25792098 10.1016/S1474-4422(15)70016-5 PMC5909703

[26] Holmes, C., C. Cunningham, E. Zotova, J. Woolford, C. Dean, S. Kerr, D. Culliford, and V. H. Perry. 2009. Systemic inflammation and disease progression in Alzheimer disease. *Neurology* 73:768–774. 10.1212/WNL.0b013e3181b6bb95. 19738171 10.1212/WNL.0b013e3181b6bb95 PMC2848584

[27] Lyman, Monty, Dafydd G.. Lloyd, Xunming Ji, Marcela P.. Vizcaychipi, and Daqing Ma. 2014. Neuroinflammation: The role and consequences. *Neuroscience Research* 79:1–12. 10.1016/j.neures.2013.10.004. 24144733 10.1016/j.neures.2013.10.004

[28] Micheau, Olivier, and Jürg. Tschopp. 2003. Induction of TNF receptor I-mediated apoptosis via two sequential signaling complexes. *Cell* 114:181–190. 10.1016/S0092-8674(03)00521-X. 12887920 10.1016/s0092-8674(03)00521-x

[29] Mishra, Anjuli, Hee Jung Kim, Angela H.. Shin, and Stanley A.. Thayer. 2012. Synapse loss induced by interleukin-1β requires pre- and post-synaptic mechanisms. *Journal of Neuroimmune Pharmacology* 7:571–578. 10.1007/s11481-012-9342-7. 22311599 10.1007/s11481-012-9342-7 PMC3415563

[30] Yang, Seung-Hoon. 2019. Cellular and molecular mediators of neuroinflammation in Alzheimer disease. *International Neurourology Journal* 23:S54-62. 10.5213/inj.1938184.092. 31795604 10.5213/inj.1938184.092 PMC6905206

[31] Leng, Fangda, and Paul Edison. 2021. Neuroinflammation and microglial activation in Alzheimer disease: Where do we go from here? *Nature Reviews Neurology* 17:157–172. 10.1038/s41582-020-00435-y. 33318676 10.1038/s41582-020-00435-y

[32] Liddelow, Shane A.., Kevin A.. Guttenplan, Laura E.. Clarke, Frederick C.. Bennett, Christopher J.. Bohlen, Lucas Schirmer, Mariko L.. Bennett, et al. 2017. Neurotoxic reactive astrocytes are induced by activated microglia. *Nature* 541:481–487. 10.1038/nature21029. 28099414 10.1038/nature21029 PMC5404890

[33] Funato, H., M. Yoshimura, T. Yamazaki, T. C. Saido, Y. Ito, J. Yokofujita, R. Okeda, and Y. Ihara. 1998. Astrocytes containing amyloid beta-protein (Abeta)-positive granules are associated with Abeta40-positive diffuse plaques in the aged human brain. *The American Journal of Pathology* 152:983–992. 9546359 PMC1858251

[34] Thal, D. R., Christian Schultz, Faramarz Dehghani, Haruyasu Yamaguchi, Heiko Braak, and Eva Braak. 2000. Amyloid β-protein (Aβ)-containing astrocytes are located preferentially near N-terminal-truncated Aβ deposits in the human entorhinal cortex. *Acta Neuropathologica* 100:608–617. 10.1007/s004010000242. 11078212 10.1007/s004010000242

[35] Ji, Kyungmin, Gulcan Akgul, Lonnie P.. Wollmuth, Stella E.. Tsirka, Anna Dunaevsky. 2013. Microglia actively regulate the number of functional synapses. *PLoS ONE* 8 : e56293. 10.1371/journal.pone.0056293. 23393609 10.1371/journal.pone.0056293 PMC3564799

[36] Hickman, Suzanne E.., Elizabeth K.. Allison, and Joseph El Khoury. 2008. Microglial dysfunction and defective β-amyloid clearance pathways in aging Alzheimer’s disease mice. *The Journal of Neuroscience* 28:8354–8360. 10.1523/JNEUROSCI.0616-08.2008. 18701698 10.1523/JNEUROSCI.0616-08.2008 PMC2597474

[37] Guerreiro, Rita, Aleksandra Wojtas, Jose Bras, Minerva Carrasquillo, Ekaterina Rogaeva, Elisa Majounie, Carlos Cruchaga, et al. 2013. *TREM2* variants in Alzheimer’s disease. *New England Journal of Medicine* 368:117–127. 10.1056/NEJMoa1211851. 23150934 10.1056/NEJMoa1211851 PMC3631573

[38] Hsieh, Christine L.., Maya Koike, Steve C.. Spusta, Erene C.. Niemi, Midori Yenari, Mary C.. Nakamura, and William E.. Seaman. 2009. A role for TREM2 ligands in the phagocytosis of apoptotic neuronal cells by microglia. *Journal of Neurochemistry* 109:1144–1156. 10.1111/j.1471-4159.2009.06042.x. 19302484 10.1111/j.1471-4159.2009.06042.x PMC3087597

[39] Frank, Stefanie, Guido J.. Burbach, Michael Bonin, Michael Walter, Wolfgang Streit, Ingo Bechmann, and Thomas Deller. 2008. TREM2 is upregulated in amyloid plaque‐associated microglia in aged APP23 transgenic mice. *Glia* 56:1438–1447. 10.1002/glia.20710. 18551625 10.1002/glia.20710

[40] Hickman, Suzanne E., Nathan D. Kingery, Toshiro K. Ohsumi, Mark L. Borowsky, Li.-chong Wang, Terry K. Means, and Joseph El Khoury. 2013. The microglial sensome revealed by direct RNA sequencing. *Nature Neuroscience* 16:1896–1905. 10.1038/nn.3554. 24162652 10.1038/nn.3554 PMC3840123

[41] Sanchez-Mejias, Elisabeth, Victoria Navarro, Sebastian Jimenez, Maria Sanchez-Mico, Raquel Sanchez-Varo, Cristina Nuñez-Diaz, Laura Trujillo-Estrada, et al. 2016. Soluble phospho-tau from Alzheimer’s disease hippocampus drives microglial degeneration. *Acta Neuropathologica* 132:897–916. 10.1007/s00401-016-1630-5. 27743026 10.1007/s00401-016-1630-5 PMC5106501

[42] Mathys, Hansruedi, Chinnakkaruppan Adaikkan, Fan Gao, Jennie Z.. Young, Elodie Manet, Martin Hemberg, Philip L.. De Jager, Richard M.. Ransohoff, Aviv Regev, and Li-Huei. Tsai. 2017. Temporal tracking of microglia activation in neurodegeneration at single-cell resolution. *Cell Reports* 21:366–380. 10.1016/j.celrep.2017.09.039. 29020624 10.1016/j.celrep.2017.09.039 PMC5642107

[43] Keren-Shaul, Hadas, Amit Spinrad, Assaf Weiner, Orit Matcovitch-Natan, Raz Dvir-Szternfeld, Tyler K.. Ulland, Eyal David, et al. 2017. A unique microglia type associated with restricting development of Alzheimer’s disease. *Cell* 169:1276-1290.e17. 10.1016/j.cell.2017.05.018. 28602351 10.1016/j.cell.2017.05.018

[44] Hampel, Harald, Filippo Caraci, A. Claudio. Cuello, Giuseppe Caruso, Robert Nisticò, Massimo Corbo, Filippo Baldacci, et al. 2020. A path toward precision medicine for neuroinflammatory mechanisms in Alzheimer’s disease. *Frontiers in Immunology* 11 : 456. 10.3389/fimmu.2020.00456. 32296418 10.3389/fimmu.2020.00456 PMC7137904

[45] Thakur, Sujata, Rishika Dhapola, Phulen Sarma, Bikash Medhi, and Dibbanti HariKrishna. Reddy. 2023. Neuroinflammation in Alzheimer’s disease: Current progress in molecular signaling and therapeutics. *Inflammation* 46:1–17. 10.1007/s10753-022-01721-1. 35986874 10.1007/s10753-022-01721-1

[46] Probert, L. 2015. TNF and its receptors in the CNS: The essential, the desirable and the deleterious effects. *Neuroscience* 302:2–22. 10.1016/j.neuroscience.2015.06.038. 26117714 10.1016/j.neuroscience.2015.06.038

[47] Breder, Christopher D.., Masafumi Tsujimoto, Yoshitake Terano, Donald W.. Scott, and Clifford B.. Saper. 1993. Distribution and characterization of tumor necrosis factor‐α‐like immunoreactivity in the murine central nervous system. *Journal of Comparative Neurology* 337:543–567. 10.1002/cne.903370403. 8288770 10.1002/cne.903370403

[48] Vitkovic, Ljubisa, Joël. Bockaert, and Claude Jacque. 2000. Inflammatory” cytokines: Neuromodulators in normal brain? *Journal of Neurochemistry* 74:457–471. 10.1046/j.1471-4159.2000.740457.x. 10646496 10.1046/j.1471-4159.2000.740457.x

[49] Boulanger, Lisa M. 2009. Immune proteins in brain development and synaptic plasticity. *Neuron* 64:93–109. 10.1016/j.neuron.2009.09.001. 19840552 10.1016/j.neuron.2009.09.001

[50] Raffaele, Stefano, Marta Lombardi, Claudia Verderio, and Marta Fumagalli. 2020. TNF production and release from microglia via extracellular vesicles: Impact on brain functions. *Cells* 9:2145. 10.3390/cells9102145. 32977412 10.3390/cells9102145 PMC7598215

[51] Ogoshi, Fumio, Hong Zhen Yin, Yuvarani Kuppumbatti, Bora Song, Simin Amindari, and John H.. Weiss. 2005. Tumor necrosis-factor-alpha (TNF-α) induces rapid insertion of Ca2+-permeable α-amino-3-hydroxyl-5-methyl-4-isoxazole-propionate (AMPA)/kainate (Ca-A/K) channels in a subset of hippocampal pyramidal neurons. *Experimental Neurology* 193:384–393. 10.1016/j.expneurol.2004.12.026. 15869941 10.1016/j.expneurol.2004.12.026

[52] Stellwagen, David, Eric C.. Beattie, Jae Y.. Seo, and Robert C.. Malenka. 2005. Differential regulation of AMPA receptor and GABA receptor trafficking by tumor necrosis factor-α. *The Journal of Neuroscience* 25:3219–3228. 10.1523/JNEUROSCI.4486-04.2005. 15788779 10.1523/JNEUROSCI.4486-04.2005 PMC6725093

[53] He, Ping, Qiang Liu, Jie Wu, and Yong Shen. 2012. Genetic deletion of TNF receptor suppresses excitatory synaptic transmission *via* reducing AMPA receptor synaptic localization in cortical neurons. *The FASEB Journal* 26:334–345. 10.1096/fj.11-192716. 21982949 10.1096/fj.11-192716 PMC3250242

[54] Leonoudakis, Dmitri, Pingwei Zhao, and Eric C.. Beattie. 2008. Rapid tumor necrosis factor α-induced exocytosis of glutamate receptor 2-lacking AMPA receptors to extrasynaptic plasma membrane potentiates excitotoxicity. *The Journal of Neuroscience* 28:2119–2130. 10.1523/JNEUROSCI.5159-07.2008. 18305246 10.1523/JNEUROSCI.5159-07.2008 PMC6671833

[55] Pribiag, Horia, and David Stellwagen. 2013. TNF-α downregulates inhibitory neurotransmission through protein phosphatase 1-dependent trafficking of GABA _A_ receptors. *The Journal of Neuroscience* 33:15879–15893. 10.1523/JNEUROSCI.0530-13.2013. 24089494 10.1523/JNEUROSCI.0530-13.2013 PMC6618471

[56] Zielinski, Mark R.., and Allison J.. Gibbons. 2022. Neuroinflammation, sleep, and circadian rhythms. *Frontiers in Cellular and Infection Microbiology* 12 : 853096. 10.3389/fcimb.2022.853096. 35392608 10.3389/fcimb.2022.853096 PMC8981587

[57] Cao, Yuan, Yali Song, Pu. Ning, Liyu Zhang, Shuang Wu, Juan Quan, and Qiao Li. 2020. Association between tumor necrosis factor alpha and obstructive sleep apnea in adults: A meta-analysis update. *BMC Pulmonary Medicine* 20 : 215. 10.1186/s12890-020-01253-0. 32787816 10.1186/s12890-020-01253-0 PMC7425010

[58] Kaushal, Navita, Vijay Ramesh, David Gozal, and Paul A.. Bartell. 2012. TNF-α and temporal changes in sleep architecture in mice exposed to sleep fragmentation. *PLoS ONE* 7 : e45610. 10.1371/journal.pone.0045610. 23029133 10.1371/journal.pone.0045610 PMC3448632

[59] Zielinski, Mark R.., Danielle L.. Dunbrasky, Ping Taishi, Gianne Souza, and James M.. Krueger. 2013. Vagotomy attenuates brain cytokines and sleep induced by peripherally administered tumor necrosis factor-α and lipopolysaccharide in mice. *Sleep* 36:1227–1238. 10.5665/sleep.2892. 23904683 10.5665/sleep.2892 PMC3700720

[60] Santello, Mirko, Paola Bezzi, and Andrea Volterra. 2011. TNFα controls glutamatergic gliotransmission in the hippocampal dentate gyrus. *Neuron* 69:988–1001. 10.1016/j.neuron.2011.02.003. 21382557 10.1016/j.neuron.2011.02.003

[61] Zhao, Huakan, Lei Wu, Guifang Yan, Yu. Chen, Mingyue Zhou, Yongzhong Wu, and Yongsheng Li. 2021. Inflammation and tumor progression: Signaling pathways and targeted intervention. *Signal Transduction and Targeted Therapy* 6 : 263. 10.1038/s41392-021-00658-5. 34248142 10.1038/s41392-021-00658-5 PMC8273155

[62] Liao, Yung-Feng., Bo-Jeng. Wang, Hui-Ting. Cheng, Lan-Hsin. Kuo, and Michael S. Wolfe. 2004. Tumor necrosis factor-α, interleukin-1β, and interferon-γ stimulate γ-secretase-mediated cleavage of amyloid precursor protein through a JNK-dependent MAPK pathway. *Journal of Biological Chemistry* 279:49523–49532. 10.1074/jbc.M402034200. 15347683 10.1074/jbc.M402034200

[63] Zhao, Jie, Tracy O’Connor, and Robert Vassar. 2011. The contribution of activated astrocytes to Aβ production: Implications for Alzheimer’s disease pathogenesis. *Journal of Neuroinflammation* 8 : 150. 10.1186/1742-2094-8-150. 22047170 10.1186/1742-2094-8-150 PMC3216000

[64] Ou, Weijun, Joshua Yang, Juste Simanauskaite, Matthew Choi, Demi M.. Castellanos, Rudy Chang, Jiahong Sun, et al. 2021. Biologic TNF-α inhibitors reduce microgliosis, neuronal loss, and tau phosphorylation in a transgenic mouse model of tauopathy. *Journal of Neuroinflammation* 18 : 312. 10.1186/s12974-021-02332-7. 34972522 10.1186/s12974-021-02332-7 PMC8719395

[65] Halliday, Glenda, Stephen R. Robinson, Claire Shepherd, and Jillian Kril. 2000. Alzheimer’s disease and inflammation: A review of cellular and therapeutic mechanisms. *Clinical and Experimental Pharmacology and Physiology* 27:1–8. 10.1046/j.1440-1681.2000.03200.x. 10696521 10.1046/j.1440-1681.2000.03200.x

[66] Wang, Shoutang, and Marco Colonna. 2019. Microglia in Alzheimer’s disease: A target for immunotherapy. *Journal of Leukocyte Biology* 106:219–227. 10.1002/JLB.MR0818-319R. 30725482 10.1002/JLB.MR0818-319R

[67] Gratuze, Maud, Cheryl E. G.. Leyns, and David M.. Holtzman. 2018. New insights into the role of TREM2 in Alzheimer’s disease. *Molecular Neurodegeneration* 13 : 66. 10.1186/s13024-018-0298-9. 30572908 10.1186/s13024-018-0298-9 PMC6302500

[68] Zhou, Mengshi, Rong Xu, David C.. Kaelber, Mark E.. Gurney, and Antony Bayer. 2020. Tumor necrosis factor (TNF) blocking agents are associated with lower risk for Alzheimer’s disease in patients with rheumatoid arthritis and psoriasis. *PLoS ONE* 15 : e0229819. 10.1371/journal.pone.0229819. 32203525 10.1371/journal.pone.0229819 PMC7089534

[69] Alvarez, Victoria, Ignacio F.. Mata, Pelayo González, Carlos H.. Lahoz, Carmen Martínez, Joaquín Peña, Luis M.. Guisasola, and Eliecer Coto. 2002. Association between the TNFα‐308 A/G polymorphism and the onset‐age of Alzheimer disease. *American Journal of Medical Genetics* 114:574–577. 10.1002/ajmg.10515. 12116197 10.1002/ajmg.10515

[70] Wang, Tengfei. 2015. TNF-alpha G308A polymorphism and the susceptibility to Alzheimer’s disease: An updated meta-analysis. *Archives of Medical Research* 46:24-30.e1. 10.1016/j.arcmed.2014.12.006. 25735998 10.1016/j.arcmed.2014.12.006

[71] Papazian, Irini, Eleni Tsoukala, Athena Boutou, Maria Karamita, Konstantinos Kambas, Lida Iliopoulou, Roman Fischer, et al. 2021. Fundamentally different roles of neuronal TNF receptors in CNS pathology: TNFR1 and IKKβ promote microglial responses and tissue injury in demyelination while TNFR2 protects against excitotoxicity in mice. *Journal of Neuroinflammation* 18 : 222. 10.1186/s12974-021-02200-4. 34565380 10.1186/s12974-021-02200-4 PMC8466720

[72] Cauwels, Anje, Ben Janssen, Anouk Waeytens, Claude Cuvelier, and Peter Brouckaert. 2003. Caspase inhibition causes hyperacute tumor necrosis factor–induced shock via oxidative stress and phospholipase A2. *Nature Immunology* 4:387–393. 10.1038/ni914. 12652297 10.1038/ni914

[73] Vandenabeele, Peter, Wim Declercq, Franky Van Herreweghe, and Tom Vanden Berghe. 2010. The role of the Kinases RIP1 and RIP3 in TNF-induced necrosis. *Science Signaling* 3. 10.1126/scisignal.3115re4. 10.1126/scisignal.3115re4 20354226

[74] Zhang, Duan-Wu., Jing Shao, Juan Lin, Na. Zhang, Bao-Ju. Lu, Sheng-Cai. Lin, Meng-Qiu. Dong, and Jiahuai Han. 2009. RIP3, an energy metabolism regulator that switches TNF-induced cell death from apoptosis to necrosis. *Science* 325:332–336. 10.1126/science.1172308. 19498109 10.1126/science.1172308

[75] Medvedev, Andrei E.., Anders Sundan, and Terje Espevik. 1994. Involvement of the tumor necrosis factor receptor p75 in mediating cytotoxicity and gene regulating activities. *European Journal of Immunology* 24:2842–2849. 10.1002/eji.1830241139. 7957575 10.1002/eji.1830241139

[76] Rao, Prakash, Katherine C.. Hsu, and Moses V.. Chao. 1995. Upregulation of NF-κB-dependent gene expression mediated by the p75 tumor necrosis factor receptor. *Journal of Interferon & Cytokine Research* 15:171–177. 10.1089/jir.1995.15.171. 8590321 10.1089/jir.1995.15.171

[77] Chen, Xin, Jeffrey J.. Subleski, Heather Kopf, O. M. Zack. Howard, Daniela N.. Männel, and Joost J.. Oppenheim. 2008. Cutting edge: Expression of TNFR2 defines a maximally suppressive subset of mouse CD4+CD25+FoxP3+ T regulatory cells: Applicability to tumor-infiltrating T regulatory cells. *The Journal of Immunology* 180:6467–6471. 10.4049/jimmunol.180.10.6467. 18453563 10.4049/jimmunol.180.10.6467 PMC2699949

[78] Tchélingérian, Jean‐Léon., Madeleine Monge, Françoise Le Saux, Bernard Zalc, and Claude Jacque. 1995. Differential oligodendroglial expression of the tumor necrosis factor receptors in vivo and in vitro. *Journal of Neurochemistry* 65:2377–2380. 10.1046/j.1471-4159.1995.65052377.x. 7595529 10.1046/j.1471-4159.1995.65052377.x

[79] Weiss, T., M. Grell, B. Hessabi, S. Bourteele, G. Müller, P. Scheurich, and H. Wajant. 1997. Enhancement of TNF receptor p60-mediated cytotoxicity by TNF receptor p80: Requirement of the TNF receptor-associated factor-2 binding site. *The Journal of Immunology* 158:2398–2404. 10.4049/jimmunol.158.5.2398. 9036990

[80] Maier, Olaf, Roman Fischer, Cristina Agresti, and Klaus Pfizenmaier. 2013. TNF receptor 2 protects oligodendrocyte progenitor cells against oxidative stress. *Biochemical and Biophysical Research Communications* 440:336–341. 10.1016/j.bbrc.2013.09.083. 24076392 10.1016/j.bbrc.2013.09.083

[81] Neta, R., T. J. Sayers, and J. J. Oppenheim. 1992. Relationship of TNF to interleukins. *Immunology Series* 56:499–566. 1550874

[82] Ortí-Casañ, Natalia, Inge S.. Zuhorn, Petrus J. W.. Naudé, Peter P.. De Deyn, Pauline E. M.. van Schaik, Harald Wajant, and Ulrich L. M.. Eisel. 2022. A TNF receptor 2 agonist ameliorates neuropathology and improves cognition in an Alzheimer’s disease mouse model. *Proceedings of the National Academy of Sciences of the United States of America* 119 : e2201137119. 10.1073/pnas.2201137119. 36037389 10.1073/pnas.2201137119 PMC9482428

[83] Almazán, Jorge Luis, Eladio Cortes-Flores, Alejandro Ramírez-Olvera, Marcela Palomero-Rivero, Nohemi Camacho-Concha, Magdalena Guerra-Crespo, G. Aleph. Prieto, Leonor Pérez-Martínez, and Gustavo Pedraza-Alva. 2025. TNFR2 contributes to synaptic potentiation failure in hippocampal synapses and memory loss in a familial Alzheimer’s disease mouse model. *Brain, Behavior, and Immunity* 129:521–536. 10.1016/j.bbi.2025.06.019. 40527437 10.1016/j.bbi.2025.06.019

[84] Carney, Brianna N.., Placido Illiano, Taylor M.. Pohl, Haritha L.. Desu, Antonella Mini, Shwetha Mudalegundi, Andoni I.. Asencor, et al. 2025. Astroglial TNFR2 signaling regulates hippocampal synaptic function and plasticity in a sex dependent manner. *Brain, Behavior, and Immunity* 129:757–777. 10.1016/j.bbi.2025.07.006. 40651585 10.1016/j.bbi.2025.07.006 PMC12323704

[85] Steeland, Sophie, Nina Gorlé, Charysse Vandendriessche, Sriram Balusu, Marjana Brkic, Caroline Van Cauwenberghe, Griet Van Imschoot, et al. 2018. Counteracting the effects of TNF receptor-1 has therapeutic potential in Alzheimer’s disease. *EMBO Molecular Medicine* 10 : e8300. 10.15252/emmm.201708300. 29472246 10.15252/emmm.201708300 PMC5887909

[86] Asimakidou, Evridiki, Richard Reynolds, Anna M.. Barron, and Chih Hung Lo. 2024. Autolysosomal acidification impairment as a mediator for TNFR1 induced neuronal necroptosis in Alzheimer’s disease. *Neural Regeneration Research* 19:1869–1870. 10.4103/1673-5374.390979. 38227498 10.4103/1673-5374.390979 PMC11040299

[87] Zhang, Nan, Ziyi Wang, and Yongxing Zhao. 2020. Selective inhibition of tumor necrosis factor receptor-1 (TNFR1) for the treatment of autoimmune diseases. *Cytokine & Growth Factor Reviews* 55:80–85. 10.1016/j.cytogfr.2020.03.002. 32327345 10.1016/j.cytogfr.2020.03.002

[88] Fischer, Roman, Jessica Marsal, Cristiano Guttà, Stephan A.. Eisler, Nathalie Peters, John R.. Bethea, Klaus Pfizenmaier, and Roland E.. Kontermann. 2017. Novel strategies to mimic transmembrane tumor necrosis factor-dependent activation of tumor necrosis factor receptor 2. *Scientific Reports* 7 : 6607. 10.1038/s41598-017-06993-4. 28747780 10.1038/s41598-017-06993-4 PMC5529482

[89] Wajant, Harald, and Daniela Siegmund. 2019. TNFR1 and TNFR2 in the control of the life and death balance of macrophages. *Frontiers in Cell and Developmental Biology* 7 : 91. 10.3389/fcell.2019.00091. 31192209 10.3389/fcell.2019.00091 PMC6548990

[90] Knoblach, Susan M.., Lei Fan, and Alan I.. Faden. 1999. Early neuronal expression of tumor necrosis factor-α after experimental brain injury contributes to neurological impairment. *Journal of Neuroimmunology* 95:115–125. 10.1016/S0165-5728(98)00273-2. 10229121 10.1016/s0165-5728(98)00273-2

[91] Longhi, Luca, Carlo Perego, Fabrizio Ortolano, Silvia Aresi, Stefano Fumagalli, Elisa R. Zanier, Nino Stocchetti, and Maria-Grazia. De Simoni. 2013. Tumor necrosis factor in traumatic brain injury: Effects of genetic deletion of p55 or p75 receptor. *Journal of Cerebral Blood Flow & Metabolism* 33:1182–1189. 10.1038/jcbfm.2013.65. 23611870 10.1038/jcbfm.2013.65 PMC3734767

[92] Yang, Jinsheng, Zerong You, Hyung-Hwan. Kim, Seo-Kyoung. Hwang, Jugta Khuman, Shuzhen Guo, Eng H. Lo, and Michael J. Whalen. 2010. Genetic analysis of the role of tumor necrosis factor receptors in functional outcome after traumatic brain injury in mice. *Journal of Neurotrauma* 27:1037–1046. 10.1089/neu.2009.1229. 20205514 10.1089/neu.2009.1229 PMC2943499

[93] Parameswaran, Narayanan, and Sonika Patial. 2010. Tumor necrosis factor-α signaling in macrophages. *Critical Reviews™ in Eukaryotic Gene Expression* 20:87–103. 10.1615/CritRevEukarGeneExpr.v20.i2.10. 21133840 10.1615/critreveukargeneexpr.v20.i2.10 PMC3066460

[94] Figiel, Izabela. 2008. Pro-inflammatory cytokine TNF-alpha as a neuroprotective agent in the brain. *Acta Neurobiologiae Experimentalis* 68:526–534. 10.55782/ane-2008-1720. 19112477 10.55782/ane-2008-1720

[95] Tanabe, Kumiko, Rie Matsushima-Nishiwaki, Shinobu Yamaguchi, Hiroki Iida, Shuji Dohi, and Osamu Kozawa. 2010. Mechanisms of tumor necrosis factor-α-induced interleukin-6 synthesis in glioma cells. *Journal of Neuroinflammation* 7 : 16. 10.1186/1742-2094-7-16. 20205746 10.1186/1742-2094-7-16 PMC2846903

[96] Rothe, Mike, Suzy C. Wong, William J. Henzel, and David V. Goeddel. 1994. A novel family of putative signal transducers associated with the cytoplasmic domain of the 75 kDa tumor necrosis factor receptor. *Cell* 78:681–692. 10.1016/0092-8674(94)90532-0. 8069916 10.1016/0092-8674(94)90532-0

[97] Rothe, Mike, Ming-Gui. Pan, William J.. Henzel, T.Merrill. Ayres, and David V.. Goeddel. 1995. The TNFR2-TRAF signaling complex contains two novel proteins related to baculoviral inhibitor of apoptosis proteins. *Cell* 83:1243–1252. 10.1016/0092-8674(95)90149-3. 8548810 10.1016/0092-8674(95)90149-3

[98] Borghi, Alice, Mira Haegman, Roman Fischer, Isabelle Carpentier, Mathieu J.M.. Bertrand, Claude Libert, Inna S.. Afonina, and Rudi Beyaert. 2018. The E3 ubiquitin ligases HOIP and cIAP1 are recruited to the TNFR2 signaling complex and mediate TNFR2-induced canonical NF-κB signaling. *Biochemical Pharmacology* 153:292–298. 10.1016/j.bcp.2018.01.039. 29378181 10.1016/j.bcp.2018.01.039

[99] Rothe, Mike, Vidya Sarma, Vishva M.. Dixit, and David V.. Goeddel. 1995. TRAF2-mediated activation of NF-κB by TNF receptor 2 and CD40. *Science* 269:1424–1427. 10.1126/science.7544915. 7544915 10.1126/science.7544915

[100] Rauert, Hilka, Andreas Wicovsky, Nicole Müller, Daniela Siegmund, Volker Spindler, Jens Waschke, Christian Kneitz, and Harald Wajant. 2010. Membrane tumor necrosis factor (TNF) induces p100 processing via TNF receptor-2 (TNFR2). *Journal of Biological Chemistry* 285:7394–7404. 10.1074/jbc.M109.037341. 20038584 10.1074/jbc.M109.037341 PMC2844188

[101] Fischer, Roman, Olaf Maier, Matthias Naumer, Anja Krippner-Heidenreich, Peter Scheurich, and Klaus Pfizenmaier. 2011. Ligand-induced internalization of TNF receptor 2 mediated by a di-leucin motif is dispensable for activation of the NFκB pathway. *Cellular Signalling* 23:161–170. 10.1016/j.cellsig.2010.08.016. 20807567 10.1016/j.cellsig.2010.08.016

[102] Sun, Shao-Cong. 2017. The non-canonical NF-κB pathway in immunity and inflammation. *Nature Reviews Immunology* 17:545–558. 10.1038/nri.2017.52. 28580957 10.1038/nri.2017.52 PMC5753586

[103] Jupp, Orla J.., Shona M.. McFARLANE, Helen M.. Anderson, Alison F.. Littlejohn, Ahmed A. A.. Mohamed, Ruth H.. Mackay, Peter Vandenabeele, and David J.. Macewan. 2001. Type II tumour necrosis factor-α receptor (TNFR2) activates c-Jun N-terminal kinase (JNK) but not mitogen-activated protein kinase (MAPK) or p38 MAPK pathways. *Biochemical Journal* 359:525–535. 10.1042/bj3590525. 11672426 10.1042/0264-6021:3590525 PMC1222173

[104] He, Tianzhen, Shuoyang Liu, Shaokui Chen, Jingyi Ye, Xueqiang Wu, Zhaoxiang Bian, and Xin Chen. 2018. The p38 MAPK inhibitor SB203580 abrogates tumor necrosis factor-induced proliferative expansion of mouse CD4+Foxp3+ regulatory T cells. *Frontiers in Immunology* 9 : 1556. 10.3389/fimmu.2018.01556. 30038619 10.3389/fimmu.2018.01556 PMC6046375

[105] Inoue, Masaki, Haruhiko Kamada, Yasuhiro Abe, Kazuma Higashisaka, Kazuya Nagano, Yohei Mukai, Yasuo Yoshioka, Yasuo Tsutsumi, and Shin-ichi Tsunoda. 2015. Aminopeptidase P3 (APP3), a novel member of the TNF/TNFR2 signaling complex, induces phosphorylation of JNK. *Journal of Cell Science**JCS* 149385. 10.1242/jcs.149385. 10.1242/jcs.149385 25609706

[106] Wang, Jun, Ricardo Ferreira, Wanhua Lu, Samatha Farrow, Kate Downes, Lutz Jermutus, Ralph Minter, Rafia S.. Al-Lamki, Jordan S.. Pober, and John R.. Bradley. 2018. TNFR2 ligation in human T regulatory cells enhances IL2-induced cell proliferation through the non-canonical NF-κB pathway. *Scientific Reports* 8 : 12079. 10.1038/s41598-018-30621-4. 30104686 10.1038/s41598-018-30621-4 PMC6089958

[107] Fischer, Roman, Olaf Maier, Martin Siegemund, Harald Wajant, Peter Scheurich, Klaus Pfizenmaier, and Malú G.. Tansey. 2011. A TNF receptor 2 selective agonist rescues human neurons from oxidative stress-induced cell death. *PLoS ONE* 6 : e27621. 10.1371/journal.pone.0027621. 22110694 10.1371/journal.pone.0027621 PMC3215731

[108] Marchetti, Lara, Matthias Klein, Katalin Schlett, Klaus Pfizenmaier, and Ulrich L.M.. Eisel. 2004. Tumor necrosis factor (TNF)-mediated neuroprotection against glutamate-induced excitotoxicity is enhanced by N-methyl-D-aspartate receptor activation. *Journal of Biological Chemistry* 279:32869–32881. 10.1074/jbc.M311766200. 15155767 10.1074/jbc.M311766200

[109] Cantley, Lewis C. 2002. The phosphoinositide 3-kinase pathway. *Science* 296:1655–1657. 10.1126/science.296.5573.1655. 12040186 10.1126/science.296.5573.1655

[110] Lawlor, Margaret A.., and Dario R.. Alessi. 2001. PKB/Akt. *Journal of Cell Science* 114:2903–2910. 10.1242/jcs.114.16.2903. 11686294 10.1242/jcs.114.16.2903

[111] Alessi, D. R. 2001. Discovery of PDKI, one of the missing links in insulin signal transduction. *Biochemical Society Transactions* 29:1–14. 10.1042/bst0290001. 11356119 10.1042/0300-5127:0290001

[112] Sarbassov, Dos D.., David A.. Guertin, Siraj M.. Ali, and David M.. Sabatini. 2005. Phosphorylation and regulation of Akt/PKB by the Rictor-mTOR complex. *Science* 307:1098–1101. 10.1126/science.1106148. 15718470 10.1126/science.1106148

[113] Griffin, Rebecca J.., Aileen Moloney, Mary Kelliher, Janet A.. Johnston, Rivka Ravid, Peter Dockery, Rosemary O’Connor, and Cora O’Neill. 2005. Activation of Akt/PKB, increased phosphorylation of Akt substrates and loss and altered distribution of Akt and PTEN are features of Alzheimer’s disease pathology. *Journal of Neurochemistry* 93:105–117. 10.1111/j.1471-4159.2004.02949.x. 15773910 10.1111/j.1471-4159.2004.02949.x

[114] Fischer, Roman, Roland Kontermann, and Olaf Maier. 2015. Targeting sTNF/TNFR1 signaling as a new therapeutic strategy. *Antibodies* 4:48–70. 10.3390/antib4010048.

[115] Jiang, Hong, Ping He, Junxia Xie, Matthias Staufenbiel, Rena Li, and Yong Shen. 2014. Genetic deletion of TNFRII gene enhances the Alzheimer-like pathology in an APP transgenic mouse model via reduction of phosphorylated I B. *Human Molecular Genetics* 23:4906–4918. 10.1093/hmg/ddu206. 24824215 10.1093/hmg/ddu206 PMC4148612

[116] McAlpine, Fiona E.., Jae-Kyung. Lee, Ashley S.. Harms, Kelly A.. Ruhn, Mathew Blurton-Jones, John Hong, Pritam Das, et al. 2009. Inhibition of soluble TNF signaling in a mouse model of Alzheimer’s disease prevents pre-plaque amyloid-associated neuropathology. *Neurobiology of Disease* 34:163–177. 10.1016/j.nbd.2009.01.006. 19320056 10.1016/j.nbd.2009.01.006 PMC2948857

[117] Paouri, Evi, Ourania Tzara, Sofia Zenelak, and Spiros Georgopoulos. 2017. Genetic deletion of tumor necrosis factor-α attenuates amyloid-β production and decreases amyloid plaque formation and glial response in the 5XFAD model of Alzheimer’s disease. *Journal of Alzheimer’s Disease* 60:165–181. 10.3233/JAD-170065. 28826177 10.3233/JAD-170065

[118] Gabbita, S. Prasad., Ming F.. Johnson, Naomi Kobritz, Pirooz Eslami, Aleksandra Poteshkina, Sridhar Varadarajan, John Turman, Frank Zemlan, Marni E.. Harris-White, and Philip C.. Trackman. 2015. Oral TNFα modulation alters neutrophil infiltration, improves cognition and diminishes tau and amyloid pathology in the 3xTgAD mouse model. *PLoS ONE* 10:e0137305. 10.1371/journal.pone.0137305. 26436670 10.1371/journal.pone.0137305 PMC4593589

[119] He, Ping, Zhenyu Zhong, Kristina Lindholm, Lilian Berning, Wendy Lee, Cynthia Lemere, Matthias Staufenbiel, Rena Li, and Yong Shen. 2007. Deletion of tumor necrosis factor death receptor inhibits amyloid β generation and prevents learning and memory deficits in Alzheimer’s mice. *The Journal of Cell Biology* 178:829–841. 10.1083/jcb.200705042. 17724122 10.1083/jcb.200705042 PMC2064547

[120] Shi, Jian-Quan., Shen Wei, Jun Chen, Bian-Rong. Wang, Ling-Ling. Zhong, Yin-Wei. Zhu, Hai-Qing. Zhu, Qiao-Quan. Zhang, Ying-Dong. Zhang, and Jun Xu. 2011. Anti-TNF-α reduces amyloid plaques and tau phosphorylation and induces CD11c-positive dendritic-like cell in the APP/PS1 transgenic mouse brains. *Brain Research* 1368:239–247. 10.1016/j.brainres.2010.10.053. 20971085 10.1016/j.brainres.2010.10.053

[121] Tweedie, David, Ryan A. Ferguson, Kelly Fishman, Kathryn A. Frankola, Henriette Van Praag, Harold W. Holloway, Weiming Luo, et al. 2012. Tumor necrosis factor-α synthesis inhibitor 3,6′-dithiothalidomide attenuates markers of inflammation, Alzheimer pathology and behavioral deficits in animal models of neuroinflammation and Alzheimer’s disease. *Journal of Neuroinflammation* 9 : 106. 10.1186/1742-2094-9-106. 22642825 10.1186/1742-2094-9-106 PMC3405480

[122] Tracey, Daniel, Lars Klareskog, Eric H.. Sasso, Jochen G.. Salfeld, and Paul P.. Tak. 2008. Tumor necrosis factor antagonist mechanisms of action: A comprehensive review. *Pharmacology & Therapeutics* 117:244–279. 10.1016/j.pharmthera.2007.10.001. 18155297 10.1016/j.pharmthera.2007.10.001

[123] Tobinick, Edward, Hyman Gross, Alan Weinberger, and Hart Cohen. 2006. TNF-alpha modulation for treatment of Alzheimer’s disease: A 6-month pilot study. *MedGenMed : Medscape General Medicine* 8 : 25. 16926764 PMC1785182

[124] Mohler, K. M., D. S. Torrance, C. A. Smith, R. G. Goodwin, K. E. Stremler, V. P. Fung, H. Madani, and M. B. Widmer. 1993. Soluble tumor necrosis factor (TNF) receptors are effective therapeutic agents in lethal endotoxemia and function simultaneously as both TNF carriers and TNF antagonists. *Journal of Immunology* 151. Baltimore. *MD* 1950:1548–1561. 8393046

[125] Feldmann, M., and R. N. Maini. 2001. Anti-TNF alpha therapy of rheumatoid arthritis: What have we learned? *Annual Review of Immunology* 19:163–196. 10.1146/annurev.immunol.19.1.163. 11244034 10.1146/annurev.immunol.19.1.163

[126] Monaco, Claudia, Jagdeep Nanchahal, Peter Taylor, and Marc Feldmann. 2015. Anti-TNF therapy: Past, present and future. *International Immunology* 27:55–62. 10.1093/intimm/dxu102. 25411043 10.1093/intimm/dxu102 PMC4279876

[127] Chou, Richard C., Michael Kane, Sanjay Ghimire, Shiva Gautam, and Jiang Gui. 2016. Treatment for rheumatoid arthritis and risk of Alzheimer’s disease: A nested case-control analysis. *CNS Drugs* 30:1111–1120. 10.1007/s40263-016-0374-z. 27470609 10.1007/s40263-016-0374-z PMC5585782

[128] Tobinick, Edward Lewis. 2016. Perispinal delivery of CNS drugs. *CNS Drugs* 30:469–480. 10.1007/s40263-016-0339-2. 27120182 10.1007/s40263-016-0339-2 PMC4920856

[129] Feldmann, Marc. 2002. Development of anti-TNF therapy for rheumatoid arthritis. *Nature Reviews. Immunology* 2:364–371. 10.1038/nri802. 12033742 10.1038/nri802

[130] Antoni, C., G. G. Krueger, K. de Vlam, C. Birbara, A. Beutler, C. Guzzo, B. Zhou, L. T. Dooley, A. Kavanaugh, and IMPACT 2 Trial Investigators. 2005. Infliximab improves signs and symptoms of psoriatic arthritis: results of the IMPACT 2 trial. *Annals of the Rheumatic Diseases* 64:1150–1157. 10.1136/ard.2004.032268. 10.1136/ard.2004.032268 PMC1755609 15677701

[131] Kaymakcalan, Zehra, Paul Sakorafas, Sahana Bose, Susanne Scesney, Limin Xiong, Denise Karaoglu Hanzatian, Jochen Salfeld, and Eric H.. Sasso. 2009. Comparisons of affinities, avidities, and complement activation of adalimumab, infliximab, and etanercept in binding to soluble and membrane tumor necrosis factor. *Clinical Immunology* 131:308–316. 10.1016/j.clim.2009.01.002. 19188093 10.1016/j.clim.2009.01.002

[132] Stübgen, Joerg-Patrick. 2008. Tumor necrosis factor-alpha antagonists and neuropathy. *Muscle & Nerve* 37:281–292. 10.1002/mus.20924. 18041052 10.1002/mus.20924

[133] MacPherson, Kathryn P.., Pradoldej Sompol, George T.. Kannarkat, Jianjun Chang, Lindsey Sniffen, Mary E.. Wildner, Christopher M.. Norris, and Malú G.. Tansey. 2017. Peripheral administration of the soluble TNF inhibitor XPro1595 modifies brain immune cell profiles, decreases beta-amyloid plaque load, and rescues impaired long-term potentiation in 5xFAD mice. *Neurobiology of Disease* 102:81–95. 10.1016/j.nbd.2017.02.010. 28237313 10.1016/j.nbd.2017.02.010 PMC5464789

[134] Barnum, Christopher J.., Xi. Chen, Jaegwon Chung, Jianjun Chang, Martha Williams, Nelly Grigoryan, Raymond J.. Tesi, and Malú G.. Tansey. 2014. Peripheral administration of the selective inhibitor of soluble tumor necrosis factor (TNF) XPro®1595 attenuates nigral cell loss and glial activation in 6-OHDA hemiparkinsonian rats. *Journal of Parkinson’s Disease* 4:349–360. 10.3233/JPD-140410. 25061061 10.3233/JPD-140410 PMC4154985

[135] Steed, Paul M.., Malú G.. Tansey, Jonathan Zalevsky, Eugene A.. Zhukovsky, John R.. Desjarlais, David E.. Szymkowski, Christina Abbott, et al. 2003. Inactivation of TNF signaling by rationally designed dominant-negative TNF variants. *Science (New York, N.Y.)* 301:1895–1898. 10.1126/science.1081297. 14512626 10.1126/science.1081297

[136] ClinicalTrials.gov. 2023. A Biomarker-directed study of XPro1595 in patients with alzheimer’s. National Library of Medicine. https://clinicaltrials.gov/ct2/show/NCT03943264

[137] Zhou, Honghui, Haishan Jang, Roy M. Fleischmann, Esther Bouman-Thio, Xu. Zhenhua, Joseph C. Marini, Charles Pendley, et al. 2007. Pharmacokinetics and safety of golimumab, a fully human anti-TNF-alpha monoclonal antibody, in subjects with rheumatoid arthritis. *Journal of Clinical Pharmacology* 47:383–396. 10.1177/0091270006298188. 17322150 10.1177/0091270006298188

[138] Shealy, David J.., Ann Cai, Kim Staquet, Audrey Baker, Eilyn R.. Lacy, Laura Johns, Omid Vafa, et al. 2010. Characterization of golimumab, a human monoclonal antibody specific for human tumor necrosis factor α. *MAbs* 2:428–439. 10.4161/mabs.12304. 20519961 10.4161/mabs.2.4.12304 PMC3180089

[139] Nesbitt, Andrew, Gianluca Fossati, Marianne Bergin, Paul Stephens, Sue Stephens, Roly Foulkes, Derek Brown, Martyn Robinson, and Tim Bourne. 2007. Mechanism of action of certolizumab pegol (CDP870): In vitro comparison with other anti-tumor necrosis factor alpha agents. *Inflammatory Bowel Diseases* 13:1323–1332. 10.1002/ibd.20225. 17636564 10.1002/ibd.20225

[140] Mancini, Francesca, Carola Marani Toro, Massimo Mabilia, Marilena Giannangeli, Mario Pinza, and Claudio Milanese. 1999. Inhibition of tumor necrosis factor-α (TNF-α)/ TNF-α receptor binding by structural analogues of suramin. *Biochemical Pharmacology* 58:851–859. 10.1016/S0006-2952(99)00150-1. 10449196 10.1016/s0006-2952(99)00150-1

[141] Alzani, R., E. Cozzi, A. Corti, M. Temponi, D. Trizio, M. Gigli, and V. Rizzo. 1995. Mechanism of suramin-induced deoligomerization of tumor necrosis factor alpha. *Biochemistry* 34:6344–6350. 10.1021/bi00019a012. 7756262 10.1021/bi00019a012

[142] He, Molly M.., Annemarie Stroustrup Smith, Johan D.. Oslob, William M.. Flanagan, Andrew C.. Braisted, Adrian Whitty, Mark T.. Cancilla, et al. 2005. Small-molecule inhibition of TNF-α. *Science* 310:1022–1025. 10.1126/science.1116304. 16284179 10.1126/science.1116304

[143] Alexiou, Polyxeni, Athanasios Papakyriakou, Evangelos Ntougkos, Christos P.. Papaneophytou, Fotini Liepouri, Anthi Mettou, Ioannis Katsoulis, et al. 2014. Rationally designed less toxic SPD-304 analogs and preliminary evaluation of their TNF inhibitory effects. *Archiv der Pharmazie* 347:798–805. 10.1002/ardp.201400198. 25160057 10.1002/ardp.201400198

[144] Naviaux, Robert K.., Brooke Curtis, Kefeng Li, Jane C.. Naviaux, A. Taylor. Bright, Gail E.. Reiner, Marissa Westerfield, et al. 2017. Low-dose suramin in autism spectrum disorder: A small, phase I/II, randomized clinical trial. *Annals of Clinical and Translational Neurology* 4:491–505. 10.1002/acn3.424. 28695149 10.1002/acn3.424 PMC5497533

[145] Cao, Yan, Ying-hua Li, Di.-ya Lv, Xiao-fei Chen, Lang-dong Chen, Zhen-yu Zhu, Yi.-feng Chai, and Jun-ping Zhang. 2016. Identification of a ligand for tumor necrosis factor receptor from Chinese herbs by combination of surface plasmon resonance biosensor and UPLC-MS. *Analytical and Bioanalytical Chemistry* 408:5359–5367. 10.1007/s00216-016-9633-6. 27225174 10.1007/s00216-016-9633-6

[146] Ruiz, Andy, Yadira Palacios, Irene Garcia, and Leslie Chavez-Galan. 2021. Transmembrane TNF and its receptors TNFR1 and TNFR2 in Mycobacterial Infections. *International Journal of Molecular Sciences* 22 : 5461. 10.3390/ijms22115461. 34067256 10.3390/ijms22115461 PMC8196896

[147] Xu, Nian-Gui., Zhi-Jie. Xiao, Ting Zou, and Zhi-Ling. Huang. 2015. Ameliorative effects of physcion 8-O-β-glucopyranoside isolated from *Polygonum cuspidatum* on learning and memory in dementia rats induced by Aβ1-40. *Pharmaceutical Biology* 53:1632–1638. 10.3109/13880209.2014.997251. 25856718 10.3109/13880209.2014.997251

[148] Lamanna, William C.., Robert Ernst Mayer, Alfred Rupprechter, Michael Fuchs, Fabian Higel, Cornelius Fritsch, Cornelia Vogelsang, et al. 2017. The structure-function relationship of disulfide bonds in etanercept. *Scientific Reports* 7 : 3951. 10.1038/s41598-017-04320-5. 28638112 10.1038/s41598-017-04320-5 PMC5479810

[149] Houel, Stephane, Mark Hilliard, Ying Qing Yu, Niaobh McLoughlin, Silvia Millan Martin, Pauline M.. Rudd, Jonathan P.. Williams, and Weibin Chen. 2014. N- and O-glycosylation analysis of etanercept using liquid chromatography and quadrupole time-of-flight mass spectrometry equipped with electron-transfer dissociation functionality. *Analytical Chemistry* 86:576–584. 10.1021/ac402726h. 24308717 10.1021/ac402726h

[150] Zalevsky, Jonathan, Thomas Secher, Sergei A.. Ezhevsky, Laure Janot, Paul M.. Steed, Christopher O’Brien, Araz Eivazi, et al. 2007. Dominant-negative inhibitors of soluble TNF attenuate experimental arthritis without suppressing innate immunity to infection. *Journal of Immunology (Baltimore, Md. : 1950)* 179:1872–1883. 10.4049/jimmunol.179.3.1872. 17641054 10.4049/jimmunol.179.3.1872

[151] Madhusudan, Srinivasan, Sethupathi R.. Muthuramalingam, Jeremy P.. Braybrooke, Susan Wilner, Kulwinder Kaur, Cheng Han, Susan Hoare, Frances Balkwill, and Trivadi S.. Ganesan. 2005. Study of etanercept, a tumor necrosis factor-alpha inhibitor, in recurrent ovarian cancer. *Journal of Clinical Oncology* 23:5950–5959. 10.1200/JCO.2005.04.127. 16135466 10.1200/JCO.2005.04.127

[152] Anfinogenova, Nina D.., Mark T.. Quinn, Igor A.. Schepetkin, and Dmitriy N.. Atochin. 2020. Alarmins and c-Jun N-terminal kinase (JNK) signaling in neuroinflammation. *Cells* 9 : 2350. 10.3390/cells9112350. 33114371 10.3390/cells9112350 PMC7693759

[153] Li, Yuanlong, Hua Fan, Ming Ni, Wei Zhang, Fengqin Fang, Jun Sun, Pin Lyu, and Peizhi Ma. 2022. Etanercept reduces neuron injury and neuroinflammation via inactivating c-Jun N-terminal kinase and nuclear factor-κB pathways in Alzheimer’s disease: An in vitro and in vivo investigation. *Neuroscience* 484:140–150. 10.1016/j.neuroscience.2021.11.001. 35058089 10.1016/j.neuroscience.2021.11.001

[154] Shih, Ruey-Horng, Chen-Yu Wang, and Chuen-Mao Yang. 2015. NF-kappaB signaling pathways in neurological inflammation: A mini review. *Frontiers in Molecular Neuroscience* 8. 10.3389/fnmol.2015.00077. 10.3389/fnmol.2015.00077 PMC4683208 26733801

[155] Ortí-Casañ, Natalia, Yingying Wu, Petrus J. W.. Naudé, Peter P.. De Deyn, Inge S.. Zuhorn, and Ulrich L. M.. Eisel. 2019. Targeting TNFR2 as a novel therapeutic strategy for Alzheimer’s Disease. *Frontiers in Neuroscience* 13 : 49. 10.3389/fnins.2019.00049. 30778285 10.3389/fnins.2019.00049 PMC6369349

[156] Yang, Mei, Jianchang Chen, Jing Zhao, and Mei Meng. 2014. Etanercept attenuates myocardial ischemia/reperfusion injury by decreasing inflammation and oxidative stress. *PLoS ONE* 9 : e108024. 10.1371/journal.pone.0108024. 25260027 10.1371/journal.pone.0108024 PMC4178063

[157] Infliximab, Infliximab-dyyb Monograph for Professionals 2019. Drugs.com. American Society of Health-System Pharmacists. Archived from the original on 15 July 2019. Retrieved 15 July 2019.

[158] Klotz, Ulrich, Alexander Teml, and Matthias Schwab. 2007. Clinical pharmacokinetics and use??of??infliximab. *Clinical Pharmacokinetics* 46:645–660. 10.2165/00003088-200746080-00002. 17655372 10.2165/00003088-200746080-00002

[159] Akiho, Hirotada, Azusa Yokoyama, Shuichi Abe, Yuichi Nakazono, Masatoshi Murakami, Yoshihiro Otsuka, Kyoko Fukawa, Mitsuru Esaki, Yusuke Niina, and Haruei Ogino. 2015. Promising biological therapies for ulcerative colitis: A review of the literature. *World Journal of Gastrointestinal Pathophysiology* 6:219–227. 10.4291/wjgp.v6.i4.219. 26600980 10.4291/wjgp.v6.i4.219 PMC4644886

[160] Shi, Jian-Quan., Bian-Rong. Wang, Wei-Wei. Jiang, Jun Chen, Yin-Wei. Zhu, Ling-Ling. Zhong, Ying-Dong. Zhang, and Jun Xu. 2011. Cognitive improvement with intrathecal administration of infliximab in a woman with Alzheimer’s disease. *Journal of the American Geriatrics Society* 59:1142–1144. 10.1111/j.1532-5415.2011.03445.x. 21668921 10.1111/j.1532-5415.2011.03445.x

[161] Kim, Dong Hyun, Seong-Min. Choi, Jihoon Jho, Man-Seok. Park, Jisu Kang, Se Jin Park, Jong Hoon Ryu, Jihoon Jo, Hyun Hee Kim, and Byeong C.. Kim. 2016. Infliximab ameliorates AD-associated object recognition memory impairment. *Behavioural Brain Research* 311:384–391. 10.1016/j.bbr.2016.06.001. 27265784 10.1016/j.bbr.2016.06.001

[162] Mohamad, Hoda E.., Dina M.. Abo-Elmatty, Nehal S.. Wahba, Mohamed A.. Shaheen, Rowan T.. Sakr, and Alaa S.. Wahba. 2022. Infliximab and/or MESNA alleviate doxorubicin-induced Alzheimer’s disease-like pathology in rats: A new insight into TNF-α/Wnt/β-catenin signaling pathway. *Life Sciences* 301 : 120613. 10.1016/j.lfs.2022.120613. 35523286 10.1016/j.lfs.2022.120613

[163] Vena, Gino A.., and Nicoletta Cassano. 2007. Drug focus: Adalimumab in the treatment of moderate to severe psoriasis. *Biologics : Targets & Therapy* 1:93–103. 19707319 PMC2721299

[164] Mease, Philip J. 2007. Adalimumab in the treatment of arthritis. *Therapeutics and Clinical Risk Management* 3:133–148. 10.2147/tcrm.2007.3.1.133. 18360621 10.2147/tcrm.2007.3.1.133 PMC1936294

[165] Scheinfeld, Noah. 2005. Adalimumab: A review of side effects. *Expert Opinion on Drug Safety* 4:637–641. 10.1517/14740338.4.4.637. 16011443 10.1517/14740338.4.4.637

[166] Highlights of Prescribing Information Humira® (adalimumab) injection, for subcutaneous use Initial U.S. Approval: 2002 https://www.accessdata.fda.gov/drugsatfda_docs/label/2021/125057s417lbl.pdf.

[167] Park, Jiae, Sun-Young. Lee, Jeeheun Shon, Koeun Kim, Hyo Jin Lee, Kyung Ah Kim, Boo-Yong. Lee, Seung-Hun. Oh, Nam Keun Kim, and Ok Joon Kim. 2019. Adalimumab improves cognitive impairment, exerts neuroprotective effects and attenuates neuroinflammation in an Aβ1-40-injected mouse model of Alzheimer’s disease. *Cytotherapy* 21:671–682. 10.1016/j.jcyt.2019.04.054. 31076196 10.1016/j.jcyt.2019.04.054

[168] Xu, Jing-Jing., Si. Guo, Rui Xue, Lin Xiao, Jun-Na. Kou, Yu-Qiong. Liu, Jun-Ya. Han, Jing-Jie. Fu, and Na. Wei. 2021. Adalimumab ameliorates memory impairments and neuroinflammation in chronic cerebral hypoperfusion rats. *Aging* 13:14001–14014. 10.18632/aging.203009. 34030135 10.18632/aging.203009 PMC8202885

[169] Steed, Paul M.., Malú G.. Tansey, Jonathan Zalevsky, Eugene A.. Zhukovsky, John R.. Desjarlais, David E.. Szymkowski, Christina Abbott, et al. 2003. Inactivation of TNF signaling by rationally designed dominant-negative TNF variants. *Science* 301:1895–1898. 10.1126/science.1081297. 14512626 10.1126/science.1081297

[170] Sama, Diana M.., Hafiz Mohmmad Abdul, Jennifer L.. Furman, Irina A.. Artiushin, David E.. Szymkowski, Stephen W.. Scheff, and Christopher M.. Norris. 2012. Inhibition of soluble tumor necrosis factor ameliorates synaptic alterations and Ca2+ dysregulation in aged rats. *PLoS ONE* 7 : e38170. 10.1371/journal.pone.0038170. 22666474 10.1371/journal.pone.0038170 PMC3362564

[171] Brambilla, Roberta, Jessica Jopek Ashbaugh, Roberta Magliozzi, Anna Dellarole, Shaffiat Karmally, David E.. Szymkowski, and John R.. Bethea. 2011. Inhibition of soluble tumour necrosis factor is therapeutic in experimental autoimmune encephalomyelitis and promotes axon preservation and remyelination. *Brain : A Journal of Neurology* 134:2736–2754. 10.1093/brain/awr199. 21908877 10.1093/brain/awr199 PMC3170538

[172] Clausen, Bettina Hjelm, Matilda Degn, Nellie Anne Martin, Yvonne Couch, Leena Karimi, Maria Ormhøj, Maria-Louise Bergholdt. Mortensen, et al. 2014. Systemically administered anti-TNF therapy ameliorates functional outcomes after focal cerebral ischemia. *Journal of Neuroinflammation* 11 : 203. 10.1186/s12974-014-0203-6. 25498129 10.1186/s12974-014-0203-6 PMC4272527

[173] Cavanagh, Chelsea, Yiu Chung Tse, Huy-Binh. Nguyen, Slavica Krantic, John C. S.. Breitner, Remi Quirion, and Tak Pan Wong. 2016. Inhibiting tumor necrosis factor-α before amyloidosis prevents synaptic deficits in an Alzheimer’s disease model. *Neurobiology of Aging* 47:41–49. 10.1016/j.neurobiolaging.2016.07.009. 27552480 10.1016/j.neurobiolaging.2016.07.009

[174] INmuneBioInc. 2019. INmune bio reports positive preliminary data from INB03 phase I clinical trial in cancer. https://www.globenewswirecom/newsrelease/2019/08/05/1896903/0/en/INmune-Bio-Reports-Positive-Preliminary-Data-from-INB03-Phase-I-Clinical-Trial-in-Cancerhtml.

[175] Barnum, Christopher, Raymond Tesi, and Jessica Malberg. 2021. Phase 1b study of XPro1595 in Alzheimer’s patients with biomarkers of inflammation. *Alzheimer’s & Dementia* 17 : e057872. 10.1002/alz.057872.

[176] Jaeger, Judith, Kim A. Staats, Sarah Barnum, Parris Pope, Lisle Kingery, Melanie Buitendyk, Sharon Cohen, Malú Gámez. Tansey, Raymond J. Tesi, and Cj. Barnum. 2025. XPro1595, a Selective Soluble TNF Neutralizer, in Early Alzheimer’s Disease with Inflammation (ADi): Results from the Phase 2 MINDFuL Trial. *Neurology*. 10.1101/2025.09.24.25336496.40315397

[177] Conference News. 2015. Systemic Inflammation: A driver of neurodegenerative disease? In.

[178] MacPherson, Kathryn P.., Lori N.. Eidson, Madelyn C.. Houser, Blaine E.. Weiss, Jenna L.. Gollihue, Mary K.. Herrick, Maria Elizabeth de Sousa Rodrigues, et al. 2023. Soluble TNF mediates amyloid-independent, diet-induced alterations to immune and neuronal functions in an Alzheimer’s disease mouse model. *Frontiers in Cellular Neuroscience* 17 : 895017. 10.3389/fncel.2023.895017. 37006470 10.3389/fncel.2023.895017 PMC10052573

[179] De Sousa Rodrigues, Maria Elizabeth, Madelyn C.. Houser, Douglas I.. Walker, Dean P.. Jones, Jianjun Chang, Christopher J.. Barnum, and Malú G.. Tansey. 2019. Targeting soluble tumor necrosis factor as a potential intervention to lower risk for late-onset Alzheimer’s disease associated with obesity, metabolic syndrome, and type 2 diabetes. *Alzheimer’s Research & Therapy* 12 : 1. 10.1186/s13195-019-0546-4. 10.1186/s13195-019-0546-4 PMC6937979 31892368

[180] Hsiao, Han-Yun., Feng-Lan. Chiu, Chiung-Mei. Chen, Yih-Ru. Wu, Hui-Mei. Chen, Yu-Chen. Chen, Hung-Chih. Kuo, and Yijuang Chern. 2014. Inhibition of soluble tumor necrosis factor is therapeutic in Huntington’s disease. *Human Molecular Genetics* 23:4328–4344. 10.1093/hmg/ddu151. 24698979 10.1093/hmg/ddu151

[181] McCoy, Melissa K.., Terina N.. Martinez, Kelly A.. Ruhn, David E.. Szymkowski, Christine G.. Smith, Barry R.. Botterman, Keith E.. Tansey, and Malú G.. Tansey. 2006. Blocking soluble tumor necrosis factor signaling with dominant-negative tumor necrosis factor inhibitor attenuates loss of dopaminergic neurons in models of Parkinson’s disease. *The Journal of Neuroscience: The Official Journal of the Society for Neuroscience* 26:9365–9375. 10.1523/JNEUROSCI.1504-06.2006. 16971520 10.1523/JNEUROSCI.1504-06.2006 PMC3707118

[182] Joers, Valerie, Gunasingh Masilamoni, Doty Kempf, Alison R.. Weiss, Travis M.. Rotterman, Benjamin Murray, Gul Yalcin-Cakmakli, et al. 2020. Microglia, inflammation and gut microbiota responses in a progressive monkey model of Parkinson’s disease: A case series. *Neurobiology of Disease* 144 : 105027. 10.1016/j.nbd.2020.105027. 32712266 10.1016/j.nbd.2020.105027 PMC7484290

[183] Xu, Zhenhua, Qingmin Wang, Yanli Zhuang, Bart Frederick, Hong Yan, Esther Bouman‐Thio, Joseph C.. Marini, et al. 2010. Subcutaneous bioavailability of golimumab at 3 different injection sites in healthy subjects. *The Journal of Clinical Pharmacology* 50:276–284. 10.1177/0091270009340782. 19940229 10.1177/0091270009340782

[184] Zhuang, Yanli, Zhenhua Xu, Bart Frederick, Dick E.. De Vries, Joyce A.. Ford, Monica Keen, Mittie K.. Doyle, Kevin J.. Petty, Hugh M.. Davis, and Honghui Zhou. 2012. Golimumab pharmacokinetics after repeated subcutaneous and intravenous administrations in patients with rheumatoid arthritis and the effect of concomitant methotrexate: An open-label, randomized study. *Clinical Therapeutics* 34:77–90. 10.1016/j.clinthera.2011.11.015. 22169051 10.1016/j.clinthera.2011.11.015

[185] Keystone, E. C., M. C. Genovese, L. Klareskog, E. C. Hsia, S. T. Hall, P. C. Miranda, J. Pazdur, et al. 2009. Golimumab, a human antibody to tumour necrosis factor α given by monthly subcutaneous injections, in active rheumatoid arthritis despite methotrexate therapy: The GO-FORWARD study. *Annals of the Rheumatic Diseases* 68:789–796. 10.1136/ard.2008.099010. 19066176 10.1136/ard.2008.099010 PMC2674549

[186] Baker, M. M., and Stephens Sue. 2006. Investigation of the pharmacokinetic properties of Certolizumab Pegol, an Anti-TNF Agent: 1117. *American Journal of Gastroenterology* 101:S437.

[187] Fleischmann, R., J. Vencovsky, R. F. Van Vollenhoven, D. Borenstein, J. Box, G. Coteur, N. Goel, H.-P. Brezinschek, A. Innes, and V. Strand. 2009. Efficacy and safety of certolizumab pegol monotherapy every 4 weeks in patients with rheumatoid arthritis failing previous disease-modifying antirheumatic therapy: The FAST4WARD study. *Annals of the Rheumatic Diseases* 68:805–811. 10.1136/ard.2008.099291. 19015206 10.1136/ard.2008.099291 PMC2674555

[188] Keystone, Edward, Désireé. Van Der Heijde, David Mason, Robert Landewé, Ronald Van Vollenhoven, Bernard Combe, Paul Emery, et al. 2008. Certolizumab pegol plus methotrexate is significantly more effective than placebo plus methotrexate in active rheumatoid arthritis: Findings of a fifty‐two–week, phase III, multicenter, randomized, double‐blind, placebo‐controlled, parallel‐group study. *Arthritis & Rheumatism* 58:3319–3329. 10.1002/art.23964. 18975346 10.1002/art.23964

[189] Smolen, Josef S.., Ronald Van Vollenhoven, Arthur Kavanaugh, Vibeke Strand, Jiri Vencovsky, Michael Schiff, Robert Landewé, et al. 2015. Certolizumab pegol plus methotrexate 5-year results from the rheumatoid arthritis prevention of structural damage (RAPID) 2 randomized controlled trial and long-term extension in rheumatoid arthritis patients. *Arthritis Research & Therapy* 17 : 245. 10.1186/s13075-015-0767-2. 26353833 10.1186/s13075-015-0767-2 PMC4565002

[190] Alzani, R., A. Corti, L. Grazioli, E. Cozzi, P. Ghezzi, and F. Marcucci. 1993. Suramin induces deoligomerization of human tumor necrosis factor alpha. *The Journal of Biological Chemistry* 268:12526–12529. 8509393

[191] Margolles-Clark, Emilio, M. Caroline. Jacques-Silva, Lakshmi Ganesan, Oliver Umland, Norma S.. Kenyon, Camillo Ricordi, Per-Olof. Berggren, and Peter Buchwald. 2009. Suramin inhibits the CD40–CD154 costimulatory interaction: A possible mechanism for immunosuppressive effects. *Biochemical Pharmacology* 77:1236–1245. 10.1016/j.bcp.2009.01.001. 19283894 10.1016/j.bcp.2009.01.001

[192] Sahu, Debasis, Ashish Saroha, Saugata Roy, Sandip Das, Prem S.. Srivastava, and Hasi R.. Das. 2012. Suramin ameliorates collagen induced arthritis. *International Immunopharmacology* 12:288–293. 10.1016/j.intimp.2011.12.003. 22178418 10.1016/j.intimp.2011.12.003

[193] Goto, T., S. Takeuchi, K. Miura, S. Ohshima, K. Mikami, K. Yoneyama, M. Sato, T. Shibuya, D. Watanabe, and E. Kataoka. 2006. Suramin prevents fulminant hepatic failure resulting in reduction of lethality through the suppression of NF-κB activity. *Cytokine* 33:28–35. 10.1016/j.cyto.2005.11.012. 16413198 10.1016/j.cyto.2005.11.012

[194] Papaneophytou, Christos P.., Vagelis Rinotas, Eleni Douni, and George Kontopidis. 2013. A statistical approach for optimization of RANKL overexpression in *Escherichia coli*: Purification and characterization of the protein. *Protein Expression and Purification* 90:9–19. 10.1016/j.pep.2013.04.005. 23623854 10.1016/j.pep.2013.04.005

[195] Melagraki, Georgia, Evangelos Ntougkos, Vagelis Rinotas, Christos Papaneophytou, Georgios Leonis, Thomas Mavromoustakos, George Kontopidis, Eleni Douni, Antreas Afantitis, and George Kollias. 2017. Cheminformatics-aided discovery of small-molecule protein-protein interaction (PPI) dual inhibitors of tumor necrosis factor (TNF) and receptor activator of NF-κB ligand (RANKL). *PLoS Computational Biology* 13 : e1005372. 10.1371/journal.pcbi.1005372. 28426652 10.1371/journal.pcbi.1005372 PMC5398486

[196] Yu, Yang, Yiling Cao, Balyssa Bell, Xiaolei Chen, Robert M.. Weiss, Robert B.. Felder, and Shun-Guang. Wei. 2019. Brain TACE (Tumor Necrosis Factor-α–Converting Enzyme) contributes to sympathetic excitation in heart failure rats. *Hypertension* 74:63–72. 10.1161/HYPERTENSIONAHA.119.12651. 31154904 10.1161/HYPERTENSIONAHA.119.12651 PMC6639045

[197] Melagraki, Georgia, Georgios Leonis, Evangelos Ntougkos, Vagelis Rinotas, Christos Papaneophytou, Thomas Mavromoustakos, George Kontopidis, Eleni Douni, George Kollias, and Antreas Afantitis. 2018. Current status and future prospects of small–molecule protein–protein interaction (PPI) inhibitors of tumor necrosis factor (TNF) and receptor activator of NF-κB ligand (RANKL). *Current Topics in Medicinal Chemistry* 18:661–673. 10.2174/1568026618666180607084430. 29875003 10.2174/1568026618666180607084430

[198] Carter, Percy H.., Peggy A.. Scherle, Jodi A.. Muckelbauer, Matthew E.. Voss, Rui-Qin. Liu, Lorin A.. Thompson, Andrew J.. Tebben, et al. 2001. Photochemically enhanced binding of small molecules to the tumor necrosis factor receptor-1 inhibits the binding of TNF-α. *Proceedings of the National Academy of Sciences* 98:11879–11884. 10.1073/pnas.211178398. 10.1073/pnas.211178398 PMC59736 11592999

